# Redox‐Mediated Stabilization of the Hole Transport Layer and Buried Interface Toward Stable Perovskite Solar Cells

**DOI:** 10.1002/anie.4012708

**Published:** 2026-03-28

**Authors:** Jiarong Wang, Yiran Yan, Chenyue Wang, Qiang Fu, Leyu Bi, Yuanzhong Liu, Xin Yang, Jia Wang, Zhenye Liang, Lin Yang, Tianjiao Chu, Xiangrong Zhu, Bin Kan, Lina Li, Xingyu Gao, Linfeng Lu, Xiaofei Ji

**Affiliations:** ^1^ Department of Materials Science and Engineering City University of Hong Kong Hong Kong P. R. China; ^2^ Shanghai Advanced Research Institute Chinese Academy of Sciences Shanghai P. R. China; ^3^ University of Chinese Academy of Sciences Beijing P. R. China; ^4^ Shanghai Synchrotron Radiation Facility (SSRF) Shanghai Institute of Applied Physics Chinese Academy of Sciences Shanghai P. R. China; ^5^ School of Energy and Materials Shanghai Polytechnic University Shanghai P. R. China; ^6^ School of Materials Science and Engineering National Institute for Advanced Materials Nankai University Tianjin P. R. China

**Keywords:** buried interface, oxalate, oxidation state regulation, perovskite solar cells, redox‐mediated

## Abstract

Achieving uniform self‐assembled monolayer (SAM) deposition on nickel oxide (NiO_x_) and suppressing interfacial defects caused by high‐oxidation‐state nickel species remains a challenge for inverted perovskite solar cells (PSCs). Here, we develop a surface modification strategy using cesium oxalate (CsOA) to synergistically regulate the NiO_x_/SAM buried interface. The CsOA treatment suppresses detrimental Ni^4+^ content and chelates with Ni^3+^ to form the complex [Ni(C_2_O_4_)_3_]^3−^, which maintains a stable oxidation state of Ni^3+^ and inhibits its continuing redox reactions as a result of the enhanced conductivity and *p*‐type characteristics. Moreover, as a buffer layer, CsOA can prevent high‐oxidation‐state nickel species (Ni^≥3+^) from reacting directly with the perovskite in uncovered regions and passivate buried perovskite defects through the interaction of the oxalate ion and under‐coordinated Pb^2+^. Additionally, the enhanced anchoring between SAM and NiO_x_/CsOA promotes uniform SAM assembly, thereby improving film quality and stability. As a result, the optimized NiO_x_/CsOA/SAM HTL enables inverted PSCs with efficiencies of 22.89% (1.67 eV) and 26.48% (1.54 eV), retaining 85.7% of the initial efficiency after 1560 h under AM1.5G illumination at 65°C. A scalable mini‐module (an active area of 11.0 cm^2^) achieves an efficiency of 23.45%, highlighting the approach's potential for high‐performance, stable, and industrially viable PSCs.

## Graphical abstract

A facile and effective strategy, that is, synergistic regulation of the NiO_x_/SAM buried interface via cesium oxalate (CsOA) modification, is developed. The CsOA treatment suppresses detrimental Ni^4+^ formation, promotes uniform SAM assembly, and aligns the energy levels at the hole‐transport layer/perovskite interface, enabling improved film crystallization and reduced interfacial defects. Inverted perovskite solar cells deliver high efficiency with enhanced device stability.

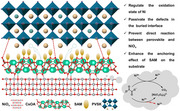

## 1 Introduction

Self‐assembled monolayers (SAMs), particularly those featuring a carbazole core and phosphonic acid functional groups such as 2PACz ([2‐(9H‐carbazol‐9‐yl)ethyl]phosphonic acid) and [4‐(3,6‐dimethyl‐9H‐carbazol‐9‐yl)butyl]phosphonic acid (Me‐4PACz), have emerged as effective hole transport layers (HTLs) in inverted perovskite solar cells (PSCs), achieving promising power conversion efficiency (PCE) [1, 2, 3, 4, 5]. These SAMs are favored for their industrial maturity and favorable optoelectronic properties [3, 6, 7, 8]. However, the interaction between SAMs and commonly used transparent conductive oxide (TCO) substrates, such as indium tin oxide (ITO) and fluorine‐doped tin oxide (FTO), often falls short of forming a uniform monolayer [9, 10, 11, 12]. Recent studies have shown that *p*‐type nickel oxide (NiO_x_) forms stronger chemical bonds with SAM molecules than ITO or FTO, allowing for more uniform SAM deposition and improved device performance and reproducibility [13, 14, 15].

Despite these advances, several limitations persist when using NiO_x_/SAM bilayer HTLs [16, 17]. For example, carbazole‐based SAMs tend to aggregate, impeding the formation of uniform dense layers on NiO_x_, which poses challenges for the scalable deposition of large‐area, high‐quality perovskite films [18, 19, 20, 21]. Moreover, the SAM layer can be desorbed by strong polar perovskite solvents such as *N,N*’‐dimethylformamide (DMF), resulting in an incomplete or loosely packed layer and exposing the underlying NiO_x_ surface to direct contact with the perovskite [1, 10, 22, 23]. Most importantly, the surface chemistry of NiO_x_ is complex, featuring Ni species in multiple oxidation states: Ni^4+^, Ni^3+^, and Ni^2+^ [24, 25]. Among these, Ni^4+^ is particularly detrimental, as it can react with A‐site organic cations in ABX_3_‐type perovskites, leading to A‐site vacancies and interfacial defects [26, 27]. Under photothermal conditions, these reactions can trigger further interfacial degradation, leading to non‐radiative recombination [28, 29]. This issue is even more pronounced in wide‐bandgap perovskites and large‐area modules, where interfacial instability can lead to phase separation and significantly compromise device stability [30, 31].

To address these challenges, recent studies have investigated strategies to enhance the quality of NiO_x_ and SAM layers [32, 33, 34]. For instance, You et al. enhanced the uniformity and conductivity of NiO_x_ by treating it with hydrogen peroxide and subsequently modifying it with Me‐4PACz, achieving inverted PSCs with over 25% efficiency and excellent stability [13]. Zhu et al. introduced MeO‐4PADBC to optimize energy alignment and strengthen the NiO_x_‐perovskite interface [14]. However, most of these efforts have focused on individual modifications of the NiO_x_ or SAM layer, without addressing the need for synergistic regulation of both sides of the buried interface [17, 35, 36]. Such an integrated approach is crucial for enhancing interfacial contact and mitigating charge accumulation at the interface.

Here, we introduce a simple yet effective surface modification strategy using cesium oxalate (CsOA) to tune the NiO_x_ surface. Note that, the chemical formula of CsOA is Cs_2_C_2_O_4_. Unlike previous studies that isolate the regulation of SAM or NiO_x_ passivation, our CsOA strategy employs redox‐mediated interface engineering to stabilize the NiO_x_/SAM buried interface synergistically, achieving comprehensive defect suppression and high stability. This approach yields three key benefits: (1) CsOA suppresses Ni^4+^ and elevates Ni^3+^ content, promoting the conductivity of NiO_x_. (2) On the modified NiO_x_/CsOA surface, Me‐4PACz assembles more uniformly due to the enhanced anchoring effect, enabling superior crystallization for uniform, large‐area films that enhance charge extraction and minimize leakage current. (3) As a buffer layer, CsOA prevents high‐oxidation‐state nickel species (Ni^≥3+^) from reacting directly with the perovskite in uncovered regions and passivates buried perovskite defects through the interaction of the oxalate ion and under‐coordinated Pb^2+^. As a result, the inverted PSCs incorporating the dual‐layer NiO_x_/CsOA/Me‐4PACz HTL achieved impressive efficiencies: 22.89% for the perovskite absorber of 1.67 eV and 26.48% for the perovskite absorber of 1.54 eV. The devices exhibited exceptional stability under the International Summit on Organic and Hybrid Photovoltaic Stability (ISOS) protocols. PSC retained 85.7% of its initial efficiency after 1560 h of operation under AM1.5G illumination at 65°C (ISOS‐L‐2I). Furthermore, a mini‐module (active area of 11.0 cm^2^) achieved a PCE of 23.45%, demonstrating the scalability and practical potential of our approach.

## 2 Results and Discussion

**FIGURE 1:**
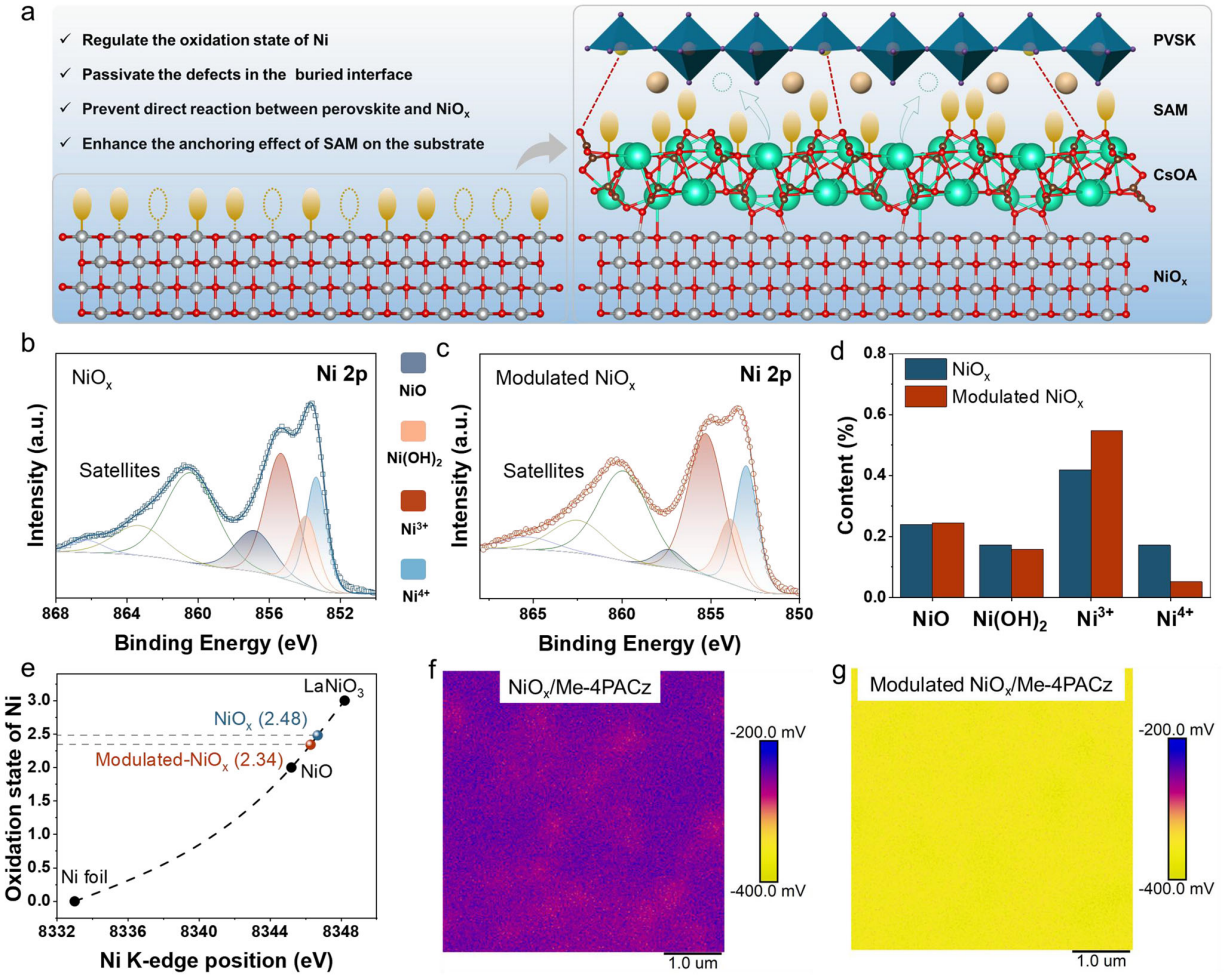
(a) Schematic diagram of the function and mechanism of the CsOA modulation in the device. XPS spectra of Ni 2p with the proportion of different valence Ni ions for (b) NiO_x_ and (c) modulated NiO_x_. (d) The variation of Ni species caused by CsOA. (e) The Ni oxidation state plotted against the Ni K‐edge position from the XANES profiles of NiO_x_ and modulated NiO_x_. The KPFM images of (f) NiO_x_/Me‐4PACz and (g) modulated NiO_x_/Me‐4PACz.

Based on the above considerations, we designed a novel cesium oxalate (CsOA) and introduced it between NiO_x_ and SAM via a layer‐by‐layer deposition strategy to enhance the overall performance of the NiO_x_/SAM interface (Figure 1a). At the mechanistic level, the oxalate anion can regulate the oxidation state of Ni, effectively reducing the content of harmful Ni^4+^ and preventing the Ni^3+^ to Ni^2+^ transition due to the strong chelating effect of oxalate ions, which increases the Ni^3+^/Ni^2+^ ratio and improves the conductivity of NiO_x_. Moreover, CsOA also acts as a buffer layer to prevent high‐oxidation‐state nickel species from reacting directly with the perovskite. Additionally, as a bidentate ligand, oxalate can coordinate with the empty d orbitals of Pb atoms, achieving passivation at the under‐coordinated Pb^2+^ sites in the buried interface of perovskite. Moreover, the incorporation of Cs^+^ further aids in passivating FA or Cs vacancies (*V*_FA_ or *V*_Cs_). Notably, the NiO_x_/CsOA interlayer not only strengthens the anchoring of SAM to the substrate, promoting its uniform deposition, but also optimizes the energy level alignment between the HTL and the perovskite. Meanwhile, CsOA forms a thin and essentially uniform interface layer on NiO_x_, promoting uniform SAM assembly, passivating exposed regions at the buried interface, and without affecting hole extraction. (Figure 1a). The redox‐mediated interface engineering strategy proposed in this work simultaneously modulates the NiO_x_ valence state, the packing density of SAMs, and the energy‐level alignment at the buried interface through precisely controlled redox reactions. This approach elegantly integrates redox‐active ligand‐based dynamic charge management with buried‐interface reconstruction, offering excellent simplicity and broad material compatibility.

Through scanning electron microscopy (SEM) images and corresponding energy dispersive spectroscopy (EDS) mapping, the CsOA distributed on the surface of nickel oxide nearly evenly and did not affect its transmittance (Figures S1 and S2). We also characterized the thickness of CsOA using atomic force microscopy (AFM), which was determined to be 4.4 nm (Figure S3). X‐ray photoemission spectroscopy (XPS) spectra were used to elucidate the variation in surface moieties resulting from CsOA treatment on NiO_x_. As shown in Figure 1b, c, the Ni 2p peaks at 853.60, 854.45, 855.60, and 856.85 eV correspond to NiO, Ni(OH)_2_, Ni^3+^, and Ni^4+^, respectively [26]. The variation in relative contents of these Ni species is plotted in Figure 1d and summarized in Table S1. CsOA‐treated NiO_x_ exhibited a higher Ni^3+^/Ni^2+^ ratio (1.36) than the pristine film (1.01), enhancing its conductivity and *p*‐type characteristics. Moreover, the content of reactive Ni^4+^, which harms the perovskite layer due to its strong oxidizing nature [37], decreases from 17.12% to 5.11%. The associated O 1s peaks (Figure S4) follow the same trend. Furthermore, x‐ray absorption near‐edge structure (XANES) analysis was conducted to probe electronic state changes in Ni within NiO_x_ and modulated NiO_x_. As shown in Figures 1e and S5, the edge position of modulated‐NiO_x_ lies between those of NiO and LaNiO_3_ based on the analysis of the first derivatives. Using established correlations between Ni oxidation state and edge energy [38, 39], the Ni oxidation states were estimated as +2.48 (NiO_x_) and +2.34 (modulated‐NiO_x_), aligning with XPS trends (+2.76 and +2.65, respectively) [27]. To elucidate these findings, we presumed the chemical reaction process as follows:

(1) $$Ni^{4+}+C_{2}{O_{4}}^{2-}\to Ni^{3+}+CO_{2}$$

(2) $$Ni^{3+}+C_{2}{O_{4}}^{2-}\to {\left[\mathrm{Ni}{\left(C_{2}O_{4}\right)}_{3}\right]}^{3-}$$

The standard electrode potential of Ni^4+^/Ni^3+^($E_{\mathrm{Ni}O_{2}/Ni_{2}O_{3}}^{\Theta }$= 1.434 V) is much larger than that of CO_2_/C_2_O_4_^2−^ ($E_{CO_{2}/C_{2}{O_{4}}^{2-}}^{\Theta }$= 0.49 V), providing the driving force for the redox reaction of Equation 1 [40]. However, the Ni^3+^ could be chelated by C_2_O_4_^2−^, as illustrated in Equation 2, maintained a stable oxidation state of Ni^3+^ and hindered its reduction to Ni^2+^, explaining the decreased Ni^4+^, increased Ni^3+^ and almost unchanged Ni^2+^ contents in modulated NiO_x_. Additionally, CsOA also effectively blocks direct contact between NiO_x_ and the perovskite as a buffer layer, thereby preventing oxidation reactions. Conductivity measurements (Figure S6) reveal values of 1.85 × 10^−6^ (pristine NiO_x_) and 3.16 × 10^−6^ S cm^−1^ (modulated NiO_x_), arising from the elevated Ni^3+^ content and verified by conductive AFM (Figure S7).

Furthermore, the altered Ni oxidation state in NiO_x_ may shift its energy levels. Ultraviolet photoelectron spectroscopy (UPS) was carried out to measure the work function (WF) of the HTL before and after modification (Figure S8). As shown in Figure S9, the WF of modulated NiO_x_/Me‐4PACz (^–^4.86 eV) is deeper than that of pristine NiO_x_/Me‐4PACz (^–^4.81 eV). Kelvin probe force microscopy (KPFM) revealed lower surface potentials for the modulated film (Figure 1f and g), consistent with the UPS results. This improved energy level alignment between HTL and perovskite promotes efficient interfacial carrier extraction, thereby boosting the open‐circuit voltage (*V*_OC_) in PSCs.

**FIGURE 2:**
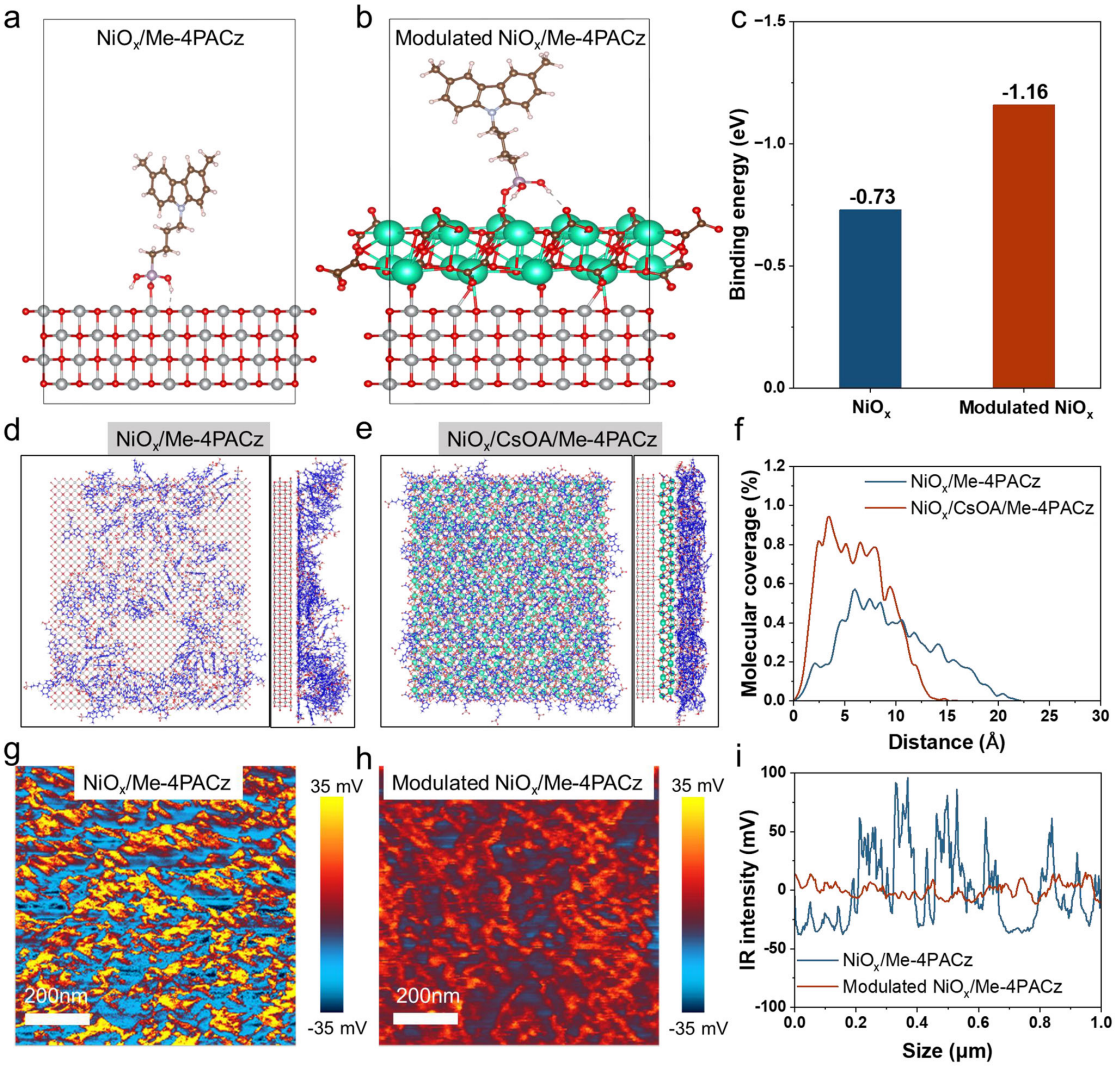
The optimized adsorption structures of Me‐4PACz at the (a) NiO_x_ and (b) modulated NiO_x_ surface. (c) Adsorption energies for SAMs at the surface of NiO_x_ and modulated NiO_x_ by CsOA. Top and side views of equilibrated molecular representations of Me‐4PACz on (d) NiO_x_/Me‐4PACz and (e) modulated NiO_x_/Me‐4PACz by simulations. (f) The changes in molecular coverages with the increasing distance between the molecular layer and the substrate. AFM‐IR morphology and 1198 cm^−1^ (P═O signal) absorption images of (g) NiO_x_/Me‐4PACz and (h) modulated NiO_x_/Me‐4PACz. (i) IR intensity data of NiO_x_ and modulated NiO_x_ films extracted from AFM‐IR images.

To investigate the impact of CsOA on the surface morphology of the SAM, we employed density functional theory (DFT) calculations to visualize the interactions between the SAM and NiO_x_ (Figure 2a, b). DFT reveals a higher binding energy for Me‐4PACz on modulated NiO_x_ (−1.16 kcal mol^−1^) than on pristine NiO_x_ (−0.73 kcal mol^−1^) (Figure 2c), indicating tighter molecular packing and enhanced interfacial contact. Additionally, molecular dynamics (MD) simulations were used to assess the aggregation of Me‐4PACz on NiO_x_ before and after modification, with a subset of SAM molecules (i.e., covalently bonded to NiO_x_) initially fixed in a vertical orientation on the substrate. As shown in Figure 2d, e, Me‐4PACz molecules prefer to aggregate on pristine NiO_x_, yielding incomplete coverage. This exposes NiO_x_ to the perovskite layer, accelerating the decomposition of amine halides in perovskite through an oxidation reaction caused by high‐valent nickel species, which leads to charge recombination and accelerated degradation at the buried interface [25]. In contrast, CsOA treatment (Figure 2f) promotes uniform Me‐4PACz distribution on NiO_x_, enhancing molecular diffusion and suppressing agglomeration to achieve homogeneous coverage. These MD insights align with atomic force microscope‐infrared (AFM‐IR) and KPFM observations. The IR signal was detected from the peak of 1198 cm^−1^, which is assigned to the bending vibrations of the P═O group in the Me‐4PACz. As shown in Figure 2g–h, the Me‐4PACz film on the pristine NiO_x_ shows a large number of highlight signals and uncovered regions, while the Me‐4PACz on the modulated NiO_x_/CsOA substrate displays a more continuous and homogeneous distribution with weaker signals and fewer exposed regions. The results suggest that, CsOA can effectively suppress aggregation and achieve uniform deposition of Me‐4PACz due to the stronger anchoring effect on the CsOA, even though some uncovered regions remain. This is confirmed by the IR intensity amplitude (Figure 2i). Furthermore, the surface potential of pristine NiO_x_/Me‐4PACz shifts markedly after washing with DMSO:DMF of 4:1 (Figure S10a), whereas modulated NiO_x_/Me‐4PACz remains stable (Figure S10b, c). This arises from stronger Me‐4PACz adsorption on modulated NiO_x_ (Figure S11). Overall, CsOA enables homogeneous Me‐4PACz distribution, bolstering interfacial binding, carrier extraction, and non‐radiative recombination suppression at the HTL/perovskite interface.

**FIGURE 3:**
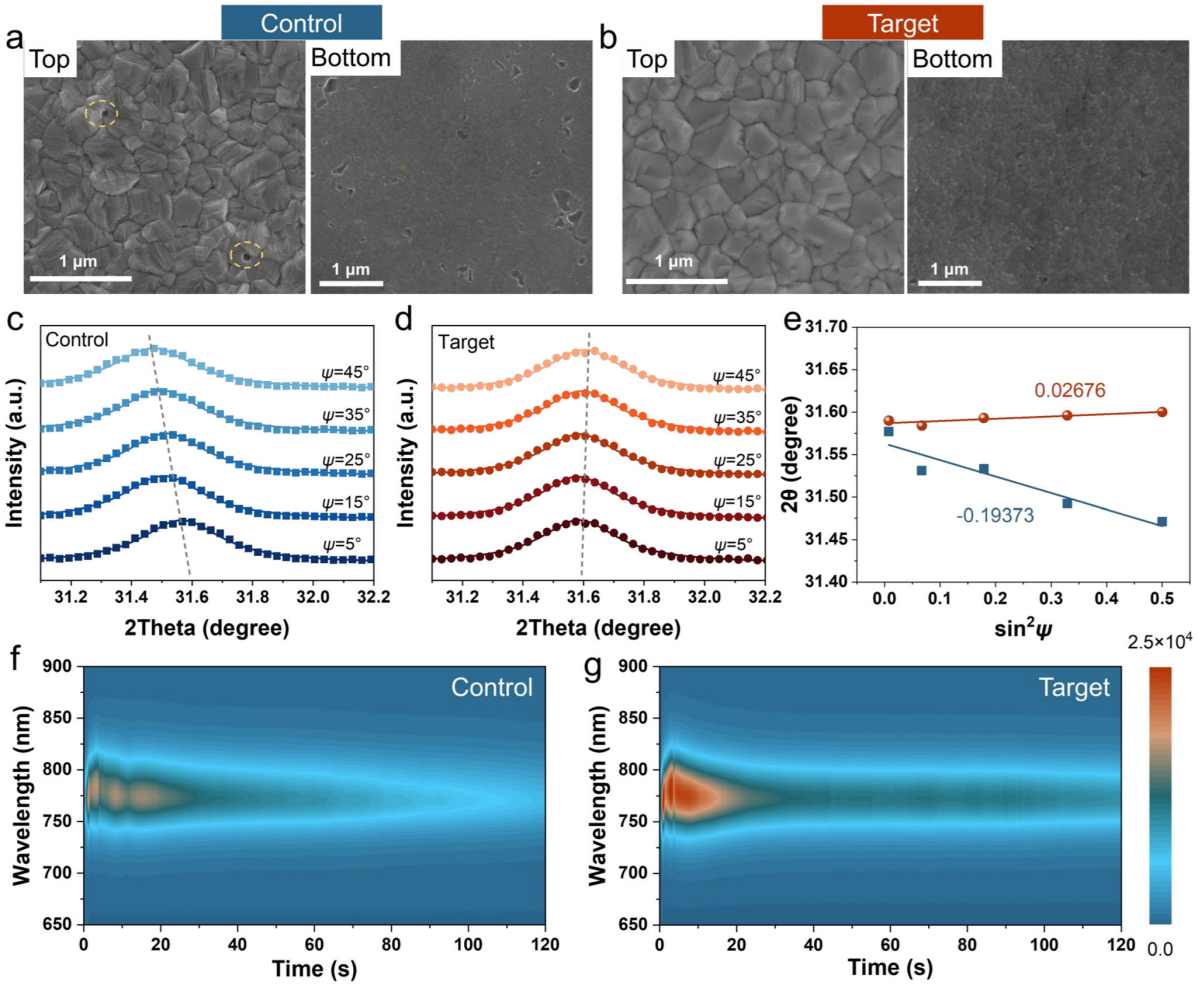
SEM images of the bottom and top surfaces for (a) control and (b) target perovskite films. GIXRD patterns measured at different *ψ* angles from 5° to 45° for (c) control and (d) target perovskite films. (e) The residual strain of the corresponding diffraction peaks (2*θ*) of control and target perovskite films as a function of sin^2^ *ψ*. Heat maps of in situ PL for (f) control and (g) target perovskite films.

To assess the impact of HTL modification on upper perovskite growth, we examined buried interfacial and surface morphologies via scanning electron microscopy (SEM) and AFM [40]. As shown in Figure 3a, b, CsOA treatment nearly eliminates nanovoids, benefiting from uniform Me‐4PACz coverage. Moreover, the perovskite film on the target HTL displays larger average grain sizes and lower root‐mean‐square roughness of 17.3 nm (Figure S12), confirming high‐quality growth. XRD analysis (Figure S13) further reveals enhanced diffraction intensity for the target perovskite compared to the control, indicating superior crystallinity, which is consistent with the SEM results. To clearly determine whether CsOA has been incorporated into the perovskite bulk phase, we prepared a FAPbI_3_ perovskite thin film. Elemental mapping reveals that, trace amounts of Cs elements have penetrated the interior of the perovskite layer in the target sample (Figure S14), confirming that a small quantity of Cs^+^ was incorporated into the perovskite lattice during the spin‐coating process.

The enhanced morphology and crystallinity at the buried perovskite interface also facilitate the release of residual stress. We quantified residual stress in perovskite films on different HTLs using grazing‐incidence x‐ray diffraction (GIXRD) with the 2*θ*‐sin^2^ *ψ* method [41]. As shown in Figure 3c, d, the (210) plane of perovskite featuring an XRD peak at 31.6° was selected as the stress‐free 2*θ* degree due to its diversity in providing more reliable structure symmetry information, in which the 2*θ* is fixed while the instrument tilt angles (*ψ*) were varied to ensure the x‐ray penetration depth [42]. Gaussian‐fitted diffraction peaks for the control perovskite shifted progressively to lower 2*θ* values as *ψ* increased from 5° to 45°. In contrast, the target perovskite exhibited a slight shift to higher 2*θ*. The slopes from linear fits of 2*θ* versus sin2*ψ* across conditions are depicted in Figure 3e. The control perovskite films exhibited negative slopes, while the target film showed a slight positive slope, indicating a shift from residual tensile stress to mild compressive stress [43]. This suggests that, CsOA modification effectively relieves residual tensile stress in perovskite films, enhancing both the efficiency and stability of PSCs.

In situ photoluminescence (PL) spectroscopy was employed to monitor the perovskite crystallization during annealing, revealing three distinct stages (Figures 3f–g and S15). In the first stage (0–10 s), corresponding to rapid nucleation and initial crystal growth [44], the target sample exhibited a markedly higher PL intensity from the onset of heating, signifying enhanced nucleation efficiency, and accelerated crystallization kinetics [45]. By comparison, the control sample displayed a more gradual response, with weaker PL signals and greater spatial dispersion. The second stage (10–20 s) involves grain boundary formation, during which PL intensity declines sharply before reaching a plateau [46]. The target sample exhibited a gentler decay and stabilized more rapidly, indicating a more ordered lattice reorganization and reduced defect generation. In the third stage (>20 s), characterized by grain ripening and further crystallization, the PL intensity of the target sample gradually recovered and sustained a higher level throughout [47]. This behavior reflects a more complete structural reconstruction, ultimately yielding perovskite films of superior quality. Therefore, the CsOA‐modified substrate significantly accelerates perovskite nucleation and promotes crystal growth, thereby effectively improving the quality of perovskite thin films.

Time‐resolved confocal fluorescence microscopy (TCFM) was employed to investigate the impact of buried interface modification on the PL intensity distribution and carrier lifetime in both control and target perovskite films. As depicted in Figure S16a, b, the target perovskite exhibits a uniform green color compared with the heterogeneous dark blue color of the control film, indicating greater PL uniformity and longer carrier lifetime. Carrier lifetime statistics from TCFM maps (Figure S16c) reveal a narrower range for the control film (54–185 ns) compared to the target film (174–425 ns). Moreover, the integrated PL lifetime counts are 2.48 × 10^6^ for the control and 7.96 × 10^6^ for the target, confirming a higher charge carrier concentration and extended lifetime in the target film. These results, consistent with steady‐state PL and time‐resolved PL (TRPL) data discussed below, underscore the superior quality of the target film, with reduced trap density and minimized non‐radiative recombination (NRR) losses.

**FIGURE 4:**
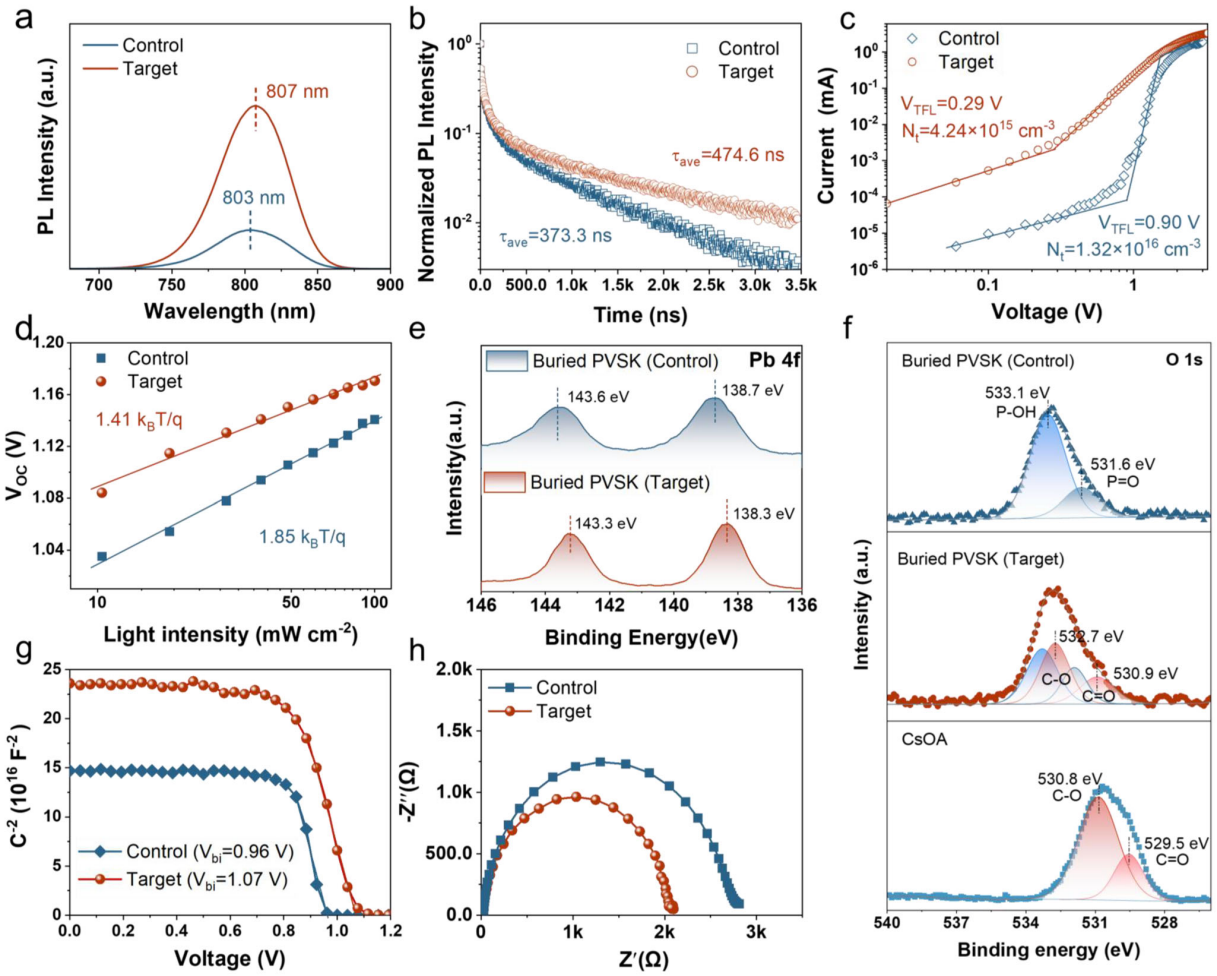
(a) Steady‐state PL spectra of perovskite films deposited on NiO_x_/Me‐4PACz and modulated NiO_x_/Me‐4PACz. (b) TRPL spectra of perovskite films deposited on NiO_x_/Me‐4PACz and modulated NiO_x_/Me‐4PACz. (c) The *I–V* curves of the hole‐only devices in the dark. (d) *V*_OC_ versus light intensity for the corresponding devices. (e) XPS spectra for Pb 4f of the control and target perovskite films at the buried interface. (f) XPS spectra for O 1s of the control and target perovskite films at the buried interface and neat CsOA films. (g) Mott–Schottky plots of the corresponding PSCs. (h) Nyquist plots for the corresponding devices.

Steady‐state PL and TRPL measurements characterized charge carrier recombination kinetics in control and target perovskite films. As shown in Figure 4a, the target film exhibits a stronger PL intensity than the control, with a slight redshift (803 to 807 nm), attributable to enhanced crystallinity [48], which is consistent with prior XRD results. Moreover, the target perovskite exhibits a longer average carrier lifetime (474.6 ns) compared to the control (373.3 ns) (Figure 4b). These elevated PL intensity and extended lifetime indicate suppressed non‐radiative recombination, likely due to the fewer buried defects in the high‐quality film, which aligns with the aforementioned observations.

We also fabricated hole‐only devices to estimate trap density (*N*_t_) and quantify defect suppression in the perovskite and at the buried interface. As shown in Figure 4c, the target perovskite exhibits a much lower *N*_t_ (4.24 × 10^15^ cm^−3^) than the control (1.32 × 10^16^ cm^−3^), attributed to its high‐quality film and fewer buried defects. To support this, recombination kinetics were examined via light intensity dependence of *V*_OC_ on a semilogarithmic scale. The target device shows a shallower slope (1.41 *k*T/*q*) than the control (1.85 *k*T/*q*), indicating reduced trap‐assisted Shockley‐Read‐Hall recombination due to CsOA modification (Figure 4d). To further elucidate suppressed NRR losses, we examined the impact of CsOA at the HTL/perovskite interface via XPS on peeled‐off buried perovskite interfaces from NiOx/Me‐4PACz (control) and modulated NiOx/Me‐4PACz (target). As shown in Figure 4e, Pb 4f and I 3d peaks shift to lower binding energies by 0.3 and 0.5 eV from control to target after CsOA modification, respectively (Figure S17). I 3d peaks shift similarly due to altered Pb‐I bonding. We attribute these perovskite peak shifts to Pb─O interactions between CsOA and uncoordinated Pb^2+^ at the buried interface. In Figure 4f, control O 1s peaks at 533.1 and 531.6 eV arise from Me‐4PACz P═O and P─OH bonds, while CsOA features C─O (530.8 eV) and C═O (529.5 eV). In the target, C─O and C═O peaks shift to higher binding energies (532.7 and 530.9 eV), with P═O and P─OH almost unchanged, confirming strong interactions between Pb^2+^ and oxalate that suppress defects at the NiOx/perovskite buried interface. Additionally, built‐in potential (*V*_bi_) was derived from capacitance‐voltage curves (Figure 4g). The target device exhibits a larger *V*_bi_ (1.07 V) than the control (0.96 V), thereby providing a stronger driving force for photogenerated charge separation and a higher *V*_OC_. Furthermore, leakage currents from the dark *J–V* curves of PSCs were also examined (Figure S18). The reverse leakage current and ideal factor (*m*) of the target devices with CsOA is smaller than that of the control, suggesting that the decreased shallow defect states and trap‐assisted Shockley–Read–Hall recombination. We attribute this to better energy level alignment and reduced defects at the HTL/perovskite interface. These results agree with those from electrochemical impedance spectroscopy, revealing a lower charge transport resistance in the target device (2017 Ω) compared to the control (2723 Ω), and confirming suppressed recombination at the interface and in the bulk (Figure 4h).

**FIGURE 5:**
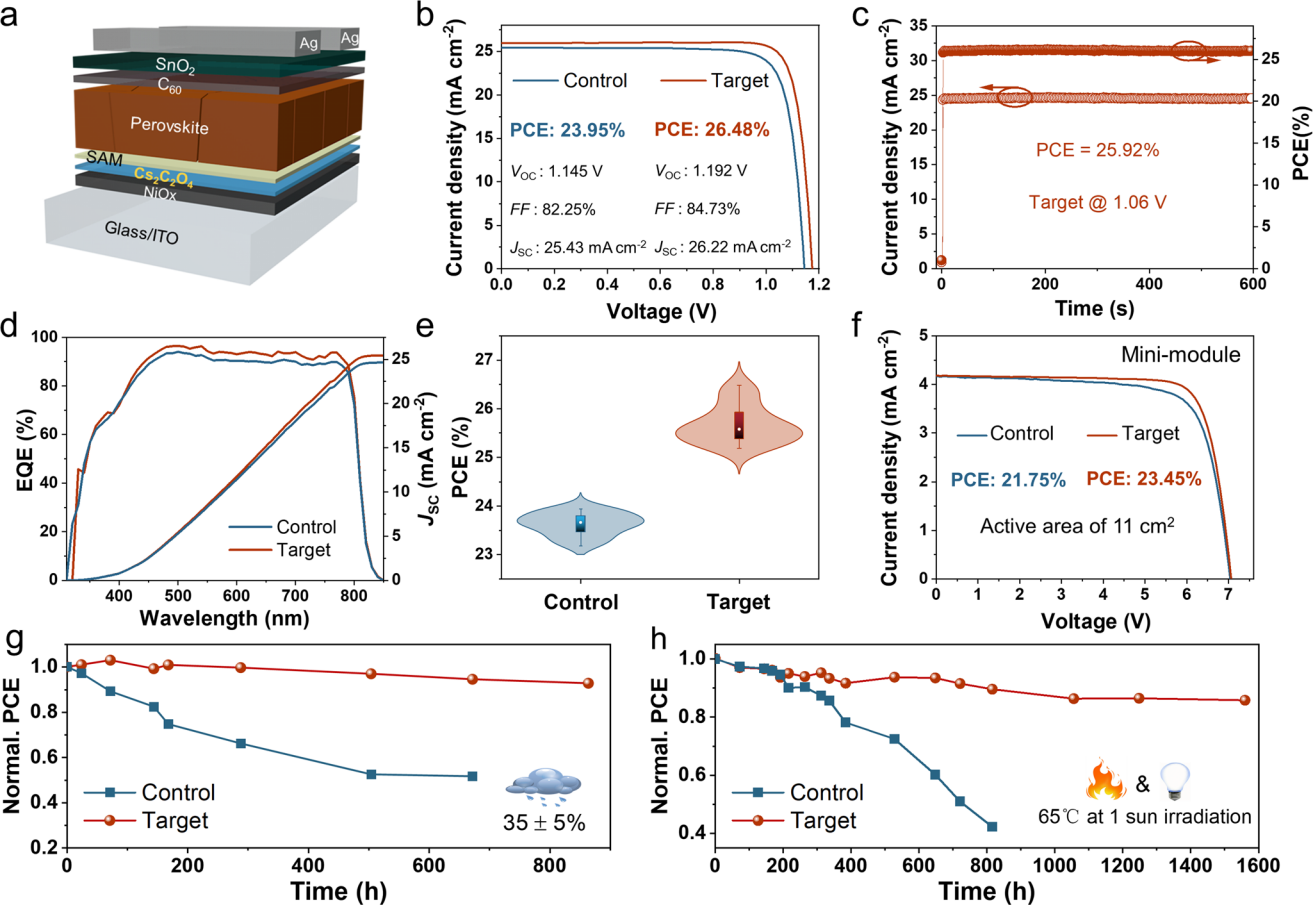
(a) The device structure of inverted PSCs. (b) The optimized *J–V* curves of the control and target devices. (c) Stabilized PCE and current density of the champion target device determined by MPP for 600 s. (d) EQE spectra with the integrated *J*_SC_ of the control and target PSCs. (e) Statistics of the PCE for the control and target devices, which are collected from 20 devices, respectively. (f) The *J–V* curves of the corresponding mini‐modules with an aperture area of 11 cm^2^. (g) Photovoltaic performance parameters of PCE as a function of time for unencapsulated devices stored in air at room temperature (RT) with a relative humidity (RH) of 35% ± 5%. (h) PCE evolution of the unencapsulated PSCs stored in the glove box at 65°C under continuous irradiation (1 sun illumination, white LED, 100 mW cm^−2^). [Correction added on 2 April 2026, after first online publication: Figure 5 has been updated.]

We fabricate inverted PSCs with the structure of ITO/NiO_x_/CsOA/Me‐4PACz/FA_0.95_Cs_0.05_PbI_3_/C_60_/SnO_2_/Ag (Figure 5a) to evaluate the device performance. The optimized current density–voltage (*J–V*) curves (Figures 5b and S19) yield a PCE of 26.48% for the target device (*V*_OC_ = 1.192 V, *J*_SC_ = 26.22 mA cm^−2^, *FF *= 84.73%; Table S2) versus 23.95% for the control (*V*_OC_ = 1.145 V, *FF *= 82.25%). These gains in *V*_OC_ and *FF* account for suppressed defects at the buried interface/bulk and improved energy alignment. Stabilized power output tracking at maximum power point shows 25.92% PCE for the target device with no decline over 600 s (Figures 5c and S20). External quantum efficiency (EQE)‐derived integrated *J*_SC_ values (24.64 mA cm^−2^ for the control and 25.45 mA cm^−2^ for the target) closely match the *J–V* results (Figure 5d). The target PSCs also demonstrate superior reproducibility, with an average PCE of 25.66% compared to 23.62% for the control (Figures 5e and S21). Performance enhancement primarily arises from elevated *V*_OC_, yielding a low bandgap voltage offset of 0.348 V for *E*_g_ = 1.54 eV (Figure S22). In addition, we explored the influence of different cations using various modifying molecules (Figure S23). The PSCs based on NiO_x_ modified with cesium iodide (CsI) or dipotassium oxalate (K_2_C_2_O_4_) achieved the PCE of 24.32% and 25.60%, respectively. These results indicate that the oxalate anion plays a more significant role in enhancing device efficiency.

To confirm that the improvement in *V*_OC_ originates from the suppression of non‐radiative recombination losses, we performed photoluminescence quantum yield (PLQY) measurements and quasi‐Fermi level splitting (QFLS) calculations on perovskite films deposited on neat NiO_x_/Me‐4PACz and modulated NiO_x_/Me‐4PACz substrates (Figure S24). The perovskite films based on the modulated NiO_x_/Me‐4PACz exhibited a notable increase in PLQY, accompanied by an increase in QFLS from 1196 mV for the control film to 1208 mV, indicating a reduction in non‐radiative recombination pathways. Moreover, the non‐radiative recombination photovoltage loss (Δ*V*_OC_,^non‐rad^) can be quantitatively derived from the measured external electroluminescence quantum efficiency (EQE_EL_) using the following equation: Δ*V*_OC_^non‐rad^ = −(kT/q)lnEQE_EL_ [49]. As shown in Figure S25, the modulated‐HTL device delivers an EQE_EL_ of 4.24% under current injection equivalent to *J*_SC_, corresponding to a Δ*V*_OC_,^non‐rad^ of 82 mV, which is significantly lower than that of the control device (100 mV). These results collectively demonstrate that, CsOA‐modulated NiO_x_/Me‐4PACz effectively decreases the defects at the HTL/perovskite interface and suppresses non‐radiative recombination, thereby contributing to the enhanced *V*_OC_. Overall, interfacial quality and tunnelling transport dominate over bulk conductivity for thin HTLs. The PCE enhancement arises from the synergistic optimization of interfacial energetics and recombination pathways, including regulated Ni oxidation states, reduced trap density, improved passivation, and suppressed non‐radiative losses, leading to concurrent increases in *FF* and *V*_OC_.

To evaluate the scalability of this method, we successfully fabricated laser‐etched perovskite solar mini‐modules (PSMs). The mini‐module with the target HTL achieved an impressive PCE of 23.45% with an active area of 11.0 cm^2^ (Figure 5f), with a *V*_OC_ of 7.065 V, a *J*_SC_ of 4.18 mA cm^−2^ and an FF of 79.37%. Additionally, a geometric fill factor (GFF) of 94% was measured for the mini‐module using a step profiler [50]. One of these mini modules was sent for independent certification to the Shanghai Institute of Microsystem and Information Technology (SIMIT, Shanghai, China), where it recorded a certified aperture‐area PCE of 21.44% (corresponding to an active‐area PCE of 22.81%) based on a reverse *J–V* scan (Figure S26). We assessed the humidity stability of unencapsulated perovskite devices at 35% ± 5% RH. As shown in Figure 5g, the target PSC retained 92.8% of its initial efficiency after 864 h of operation. In contrast, the control retained only 51.7% of its original value after 675 h of operation. Additionally, under continuous one‐sun illumination (white LED) at 65°C in N_2_, the unencapsulated target device maintained 85.7% of its initial PCE after 1560 h, compared to 42.3% for the control after 816 h (Figure 5h). The enhanced stability mainly results from suppressing the defects at the buried interface or bulk. Moreover, CsOA also acted as a buffer layer to block the oxidation reaction between high‐valent nickel species and the perovskite, thereby slowing down the degradation of the perovskite.

**FIGURE 6:**
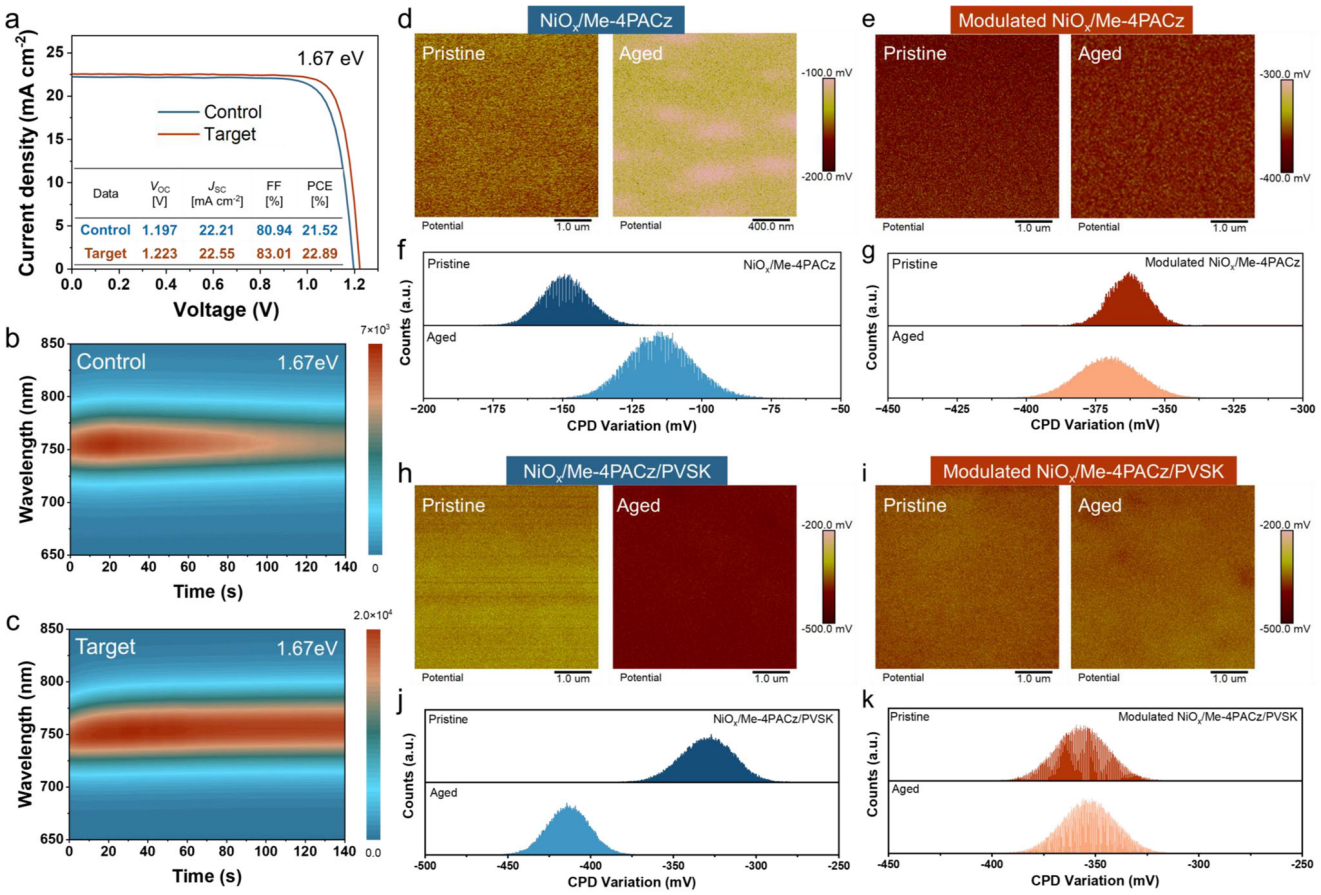
(a) The optimized *J–V* curves of the control and target devices with the bandgap of 1.67 eV. PL stability in an air environment of (b) control and (c) target film with 1.67 eV. Surface potential images obtained by scanning Kelvin probe microscopy of (d) NiO_x_/Me‐4PACz and (e) modulated NiO_x_/Me‐4PACz films before and after 48 h 65°C thermal heating and 1‐sun light exposure in N_2_ environment. Statistical potential distribution of (f) NiO_x_/Me‐4PACz and (g) modulated NiO_x_/Me‐4PACz films extracted from the top figures. Surface potential images obtained by scanning Kelvin probe microscopy of the perovskite films on the (h) NiO_x_/Me‐4PACz and (i) modulated NiO_x_/Me‐4PACz before and after 48 h 65°C thermal heating and 1‐sun light exposure in N_2_ environment. Statistical potential distribution of the perovskite films on the (j) NiO_x_/Me‐4PACz and (k) modulated NiO_x_/Me‐4PACz extract from the top figures. [Correction added on 2 April 2026, after first online publication: In caption of Figure 6a “1.68 eV” has been corrected to “1.67 eV”.]

Moreover, we assessed the universality of our strategy using wide‐bandgap perovskite absorbers (1.67 eV). As shown in Figure 6a, the optimized device achieves a PCE of 22.89% with a high *V*_OC_ of 1.223 V, an *FF* of 83.01%, and a *J*_SC_ of 22.55 mA cm^−2^. Notably, the modified perovskite exhibits exceptional stability under continuous irradiation. PL intensity under 512 nm laser excitation (Figure 6b, c) reveals a higher initial PL for the target film than for the control, with the target retaining 91% of its initial intensity after 140 s, compared to 66% for the control. We further employed KPFM to assess the stability of HTL and perovskite films. As shown in Figure 6d and f, the control HTL's surface potential increased from ─150 to ─115 mV after 48 h at 65°C under one sun illumination, with a broad contact potential difference (CPD) distribution. In contrast, the target HTL exhibited negligible CPD shift from ─363 to ─370 mV (Figure 6e and g). Similarly, perovskites on the modulated HTL showed superior stability. The control perovskite's surface potential decreased from ─328 to ─413 mV post‐aging (Figure 6h and j). In contrast, the target perovskite's CPD varied insignificantly from ─356 to ─352 mV (Figure 6i and k). The introduction of CsOA not only enhances the interfacial bonding between the HTL and perovskite, but also significantly optimizes the HTL/perovskite buried interface, promoting the deposition of high‐quality perovskite films and acting as a buffer layer against direct contact between NiO_x_ and perovskite, thereby improving the overall stability of the HTL/perovskite film.

## 3 Conclusion

In summary, this work addresses the key challenges in NiO_x_/SAM bilayer HTLs for inverted PSCs by introducing a facile CsOA surface modification strategy that synergistically suppresses Ni^4+^ species, enhances SAM uniformity, prevents the direct reaction between high‐valent nickel species and perovskite, and passivates buried defects. The resulting optimized HTL yields remarkable PCEs of 22.89% (1.67 eV wide‐bandgap perovskite) and 26.48% (1.54 eV bandgap perovskite), with devices retaining 85.7% of their initial efficiency after 1560 h under AM1.5G illumination at 65°C (ISOS‐L‐2I), alongside a scalable mini module PCE of 23.45% with an active area of 11.0 cm^2^. These advancements underscore the potential of integrated interface engineering for high‐performance and stable PSCs.

## Conflicts of Interest

The authors declare no conflicts of interest.

## Supporting information




**Supporting File 1**: anie71975‐sup‐0001‐SuppMat.docx.

## Data Availability

The data that support the findings of this study are available from the corresponding author upon reasonable request.
