# Supplementary material for: Redox‐Mediated Stabilization of the Hole Transport Layer and Buried Interface Toward Stable Perovskite Solar Cells
Source: Angew Chem Int Ed Engl. 2026 Mar 28;65(19):e4012708. doi: 10.1002/anie.4012708 (PMC13134593; doi:10.1002/anie.4012708)
Supplement: Supplementary file 1 — Supporting File 1: anie71975‐sup‐0001‐SuppMat.docx. [file ANIE-65-e4012708-s001.docx]

Supporting Information
©Wiley-VCH 2021
69451 Weinheim, Germany

**Redox-Mediated Stabilization of the Hole Transport Layer and Buried Interface toward Stable Perovskite Solar Cells**

Jiarong Wang^#^, Yiran Yan^#^, Chenyue Wang^#^, Qiang Fu*, Leyu Bi, Yuanzhong Liu, Xin Yang, Jia Wang, Zhenye Liang, Lin Yang, Tianjiao Chu, Xiangrong Zhu, Bin Kan, Lina Li, Xingyu Gao*, Linfeng Lu* and Xiaofei Ji*

**Materials**

NiO_x_ nanoparticles (99.999%), formamidinium iodide (FAI, 99.5%), lead iodide (PbI_2_, 99.999%), lead(II) bromide(PbBr_2_, 99.999%), cesium iodide (CsI, 99.999%), cesium oxalate (CsOA) and methylamine hydrochloride (MACl, 99.9%) were purchased from Advanced Election Technology Co., Ltd, N,N-dimethylformamide (DMF, 99.8%), dimethyl sulfoxide (DMSO, 99.5%), anisole (99%), ethanol (99.5%), isopropanol (IPA, 99.5%) and chlorobenzene (CB, 99.8%) were purchased from Sigma-Aldrich. Ethylenediamine dihydroiodide (EDAI_2_, ≥98%), lead thiocyanate (Pb(SCN)_2_, 99.5%), Piperazinium iodide (PI, 99.5%) and buckminsterfullerene (C_60_, 99.5%) were purchased from Xi’an Yuri Solar. [4-(3,6-dimethyl-9H-carbazol-9-yl)butyl]phosphonic acid (Me-4PACz, >99.0%) was purchased from TCI.

**Perovskite precursor and film preparation (1.54 eV)**

For the composition FA_0.95_Cs_0.05_PbI_3_, 1.5 M perovskite precursor solution was prepared by mixing CsI, FAI and PbI_2_ in DMF: DMSO (4:1/v:v) mixed solvent subject to the stochiometric ratio. An additional 3 mol% PbI_2_ and 10 mol% MACl were added to the precursor for better crystallization and perovskite phase transformation. The precursor was stirred overnight at room temperature in a N_2_-filled glovebox and filtered through a 0.22 μm polytetrafluoroethylene (PTFE) filter before use. The perovskite precursor (50 μl) was spin-coated at 4000 rpm for 50 s (5 s acceleration to 4000 rpm). CB (200 μl) was dropped on the film 20 s before the end of the spinning. The film was immediately annealed at 100 °C for 30 min.

**Small-area devices fabrication (1.54 eV)**

The glass/ITO substrate was cleaned by sequential ultrasonication for 30 min with isopropanol, acetone and isopropanol. Before use, the substrate was cleaned with ultraviolet ozone for 20 min. The substrate was spin-coated with a thin layer of NiO_x_ nanoparticles film (10 mg ml^-1^ NiO_x_ in deionized water) at 2000 rpm for 20 s, annealed at 150 °C for 10 min in ambient air and then transferred to a N_2_-filled glovebox. Subsequently, for the control device, Me-4PACz (0.5 mg ml^-1^ in ethanol) was deposited on the NiO_x_ at 3000 rpm for 30 s, followed by annealing at 100 °C for 10 min. For the target device, the CsOA (3.5 mg ml^-1^ in deionized water) was spin-coated on the NiO_x_ at 3000 rpm for 30 s, followed by annealing at 100 °C for 10 min. The perovskite film was spin-coated on NiO_x_/Me-4PACz (or NiO_x_/CsOA/Me-4PACz ) following the abovementioned method. For the interfacial passivation layer, PI (0.2 mg ml^-1^ in IPA) was spin-coated on the surface of the perovskite film at 3000 rpm for 30 s and annealed at 100 °C for 10 min. After that, 20 nm C_60_ was thermally evaporated on top of the perovskite layer. 10 nm SnO_2_ was then deposited using the thermal atomic layer deposition (ALD) technique. Finally, 130 nm thickness of Ag was thermally evaporated as an electrode using a shadow mask.

**Fabrication of mini-modules**

To prepare the perovskite solar modules, the washed ITO substrates (5 × 5 cm^2^) with laser-etched P1 (25 μm wide) were treated with UV-ozone for 20 min. The substrate was spin-coated with a thin layer of NiO_x_ nanoparticles film (10 mg ml^-1^ NiO_x_ in deionized water) at 2000 rpm for 20 s, annealed at 150 °C for 10 min in ambient air and then transferred to a N_2_-filled glovebox. Subsequently, for the control device, Me-4PACz (0.5 mg ml^-1^ in ethanol) was deposited on the NiO_x_ at 3000 rpm for 30 s, followed by annealing at 100 °C for 10 min. For the target device, the CsOA (3.5 mg ml^-1^ in deionized water) was spin-coated on the NiO_x_ at 3000 rpm for 30 s, followed by annealing at 100 °C for 10 min. Then, Me-4PACz was deposited.

150 μL perovskite precursor was spin-coated at 2,000 rpm for 10 s and 5,000 rpm for 80 s on NiO_x_/Me-4PACz (or NiO_x_/CsOA/Me-4PACz ); 550 μL CB as the antisolvent was dripped on the film at 10 s before the end of the spin procedure and then annealed at 100 °C for 30 min. As for the passivation layer, PI (0.25 mg/mL in IPA) is spin-coated on the perovskite film at 3000 rpm for 30 s, then annealed at 100 ^o^C for 10 min. The films were then cooled to room temperature and ready for thermal evaporation. Subsequently, 25 nm C_60_ and 6 nm BCP were sequentially evaporated under a high vacuum (<5 × 10^-6^ torr). After that, P2 (125 μm wide) was etched by laser. Then, 100 nm Ag was evaporated as the electrode. Finally, P3 (65 μm wide) was etched by laser. GFF is about 94%. For the anti-reflection coating, an MgF_2_ layer with a thickness of 140 nm was thermally evaporated onto the back of the devices.

**Perovskite precursor and film preparation (1.67 eV)**

For the composition FA_0.8_Cs_0.2_Pb(I_0.8_Br_0.2_)_3_, 1M perovskite precursor solution was prepared by mixing FAI, CsI, PbI_2_ and PbBr_2_ in DMF: DMSO (3:1/v:v) mixed solvent subject to the stochiometric ratio. Then, 6.46 mg (2 mol% relative to Pb) Pb(SCN)_2_ was added to the solution. The perovskite precursor solution was thoroughly mixed and aged for 12 hours before use.

**Small-area devices fabrication (1.67 eV)**

The glass/ITO substrate was cleaned by sequential ultrasonication for 30 min with isopropanol, acetone and isopropanol. Before use, the substrate was cleaned with ultraviolet ozone for 20 min. The substrate was spin-coated with a thin layer of NiO_x_ nanoparticles film (10 mg ml^-1^ NiO_x_ in deionized water) at 2000 rpm for 20 s, annealed at 150 °C for 10 min in ambient air and then transferred to a N_2_-filled glovebox. Subsequently, for the control device, Me-4PACz (0.5 mg ml^-1^ in ethanol) was deposited on the NiO_x_ at 3000 rpm for 30 s, followed by annealing at 100 °C for 10 min. The WBG perovskite was spin-coated at 500 rpm 2 s and 4000 rpm 60s with 200 μL anisole dripped slowly at 25 s in the second spinning step. We annealed the as-deposited films at 65 °C for 5 min and 100 °C for 15 min. The 0.5 mg ml^-1^ EDAI_2_ solution (dissolved in IPA) was spin-coated on the perovskite top surface at 5000 rpm for 30 s and then annealed at 100 °C for 5 min or dried at room temperature. After that, 20 nm C_60_ was thermally evaporated on top of the perovskite layer. 10 nm SnO_2_ was then deposited using the thermal atomic layer deposition (ALD) technique. Finally, 130 nm thickness of Ag was thermally evaporated as an electrode using a shadow mask.

**Measurements and Instruments**

Cyclic voltammetry measurements were conducted using a CHI1020D electrochemical workstation. The experiments were carried out at room temperature employing a conventional three-electrode system. This system consisted of a glassy carbon electrode as the working electrode, Pt wire as the counter electrode, and Ag/AgCl (saturated KCl) as the reference electrode. To obtain cyclovoltagrams, the powder was dissolved in 0.5 M Na_2_SO_4_ aqueous electrolyte solution. X-ray absorption near-edge structure (XANES) was performed at the beamline BL16U1 of the Shanghai Synchrotron Radiation Facility (SSRF). For Grazing incidence X-ray diffraction (GIXRD), the (210) plane of perovskite featuring an XRD peak at 31.6° was selected as the stress-free 2θ degree due to its diversity in providing more reliable structure symmetry information, in which the 2θ is fixed while the instrument tilt angles were varied to ensure the X-ray penetration depth. The tilt angles (ψ) were fixed at 5°, 15°, 25°, 35°, and 45°, respectively. Scanning electron microscopic (SEM) images were obtained by ZEISS Gemini300 (Carl Zeiss Microscopy Co. Ltd.). Ultraviolet-visible absorption spectra were characterized on a spectrophotometer (Cary 60 UV-Vis, Agilent). Steady-state photoluminescence (PL) was recorded by the Edinburgh FLS920 fluorescence spectrometer (Edinburgh) with an excitation wavelength of 450 nm. Time-resolved photoluminescence (TRPL) was measured using Fluorolog-3 (Edinburgh Instruments Ltd.) with an excitation wavelength of 450 nm. XPS measurements were performed on a Thermo Fisher Scientific Nexsa using a monochromatic Al Kα (1486.6 eV) radiation. A 500μm X-ray spot was used for XPS analysis. Typically, the hydrocarbon C1s line at 284.8 eV from adventitious carbon was used for energy referencing. UPS measurements were also investigated by a Thermo Fisher Scientific Nexsa, with the He Iα (21.22 eV) emission line employed for excitation.

**Space-charge-limited current (SCLC) measurements**

The hole-only devices for the SCLC method were fabricated with the structure of ITO/NiO_x_/Me-4PACz/perovskite/PTAA/Ag or ITO/NiO_x_/CsOA/Me-4PACz /perovskite/PTAA/Ag. The dark I-V curves were obtained using a Keithley 2400 Source Meter. The trap-state density of perovskite films can be calculated as follows:

$$N_{trap}=\frac{2V_{TFL}\varepsilon_{r}\varepsilon_{0}}{qL^{2}}$$

Where *N_trap_* is the trap-state density, *V_TFL_* is the trap-filled limit voltage, *ε_r_* is the relative dielectric constant for the perovskite film, *ε_0_* is the vacuum permittivity (8.854×10^-12^ F m^-1^), *q* is the electric charge (1.6×10^-19^ C), and *L* is the thickness of the perovskite film (~550 nm).

Calculation of quasi-Fermi level splitting (QFLS) and non-radiative recombination photovoltage loss (Δ*V*_OC_^non-rad^)

QFLS can be calculated from PLQY by the following formula:

*QFLS* = *QFLS_rad_* + *k_B_T*ln(*PLQY*)

Where, QFLS_rad_ is the radiation limit of semiconductor materials, which sets the maximum achievable splitting of the quasi-Fermi level without considering non-radiative recombination.

From that, the QFLS_rad_ (PLQE = 100%) can be calculated with the following equation:

*QFLS_rad_* = *k_B_Tln*($\frac{J_{G}}{J_{0, rad}}$)

*k*_B_ is the Boltzmann constant, *J*_G_ is the photogenerated current density, *J*_0, rad_ is the dark state radiative recombination saturation current density. In this case, we approximated *J*_G_ to short circuit current J_SC_ and used the room temperature where T=300 K. As such, we calculated the QFLS_rad_ of our perovskite absorber as:

*QFLS_rad_ = k_B_Tln*($\frac{J_{G}}{J_{0, rad}}$)=1.276 eV

**Device Characterizations**

Current density-voltage (*J-V*) curves were measured using a Keithley 2400 Source Meter under simulated AM1.5G illumination (Enli SS-F5-3A; Enli Technology, Taiwan), and the light intensity was calibrated using a standard silicon reference cell (Enli Technology Co., Ltd., Taiwan).

**Preparation of the buried perovskite interface.**

The buried interface was obtained by exfoliating the perovskite film from the glass/ITO/SnO_2_ substrate. The specific method is described below: UV curable glue was applied on the surface of the perovskite film after thermal annealing, which was then covered with glass. The samples were then placed in a UV lamp box (350 W) for 1 min to cure the UV adhesive. Finally, two tweezers were used to clamp the substrate and glass, and the perovskite film was then peeled off the substrate.

**Supporting Figures.**

**
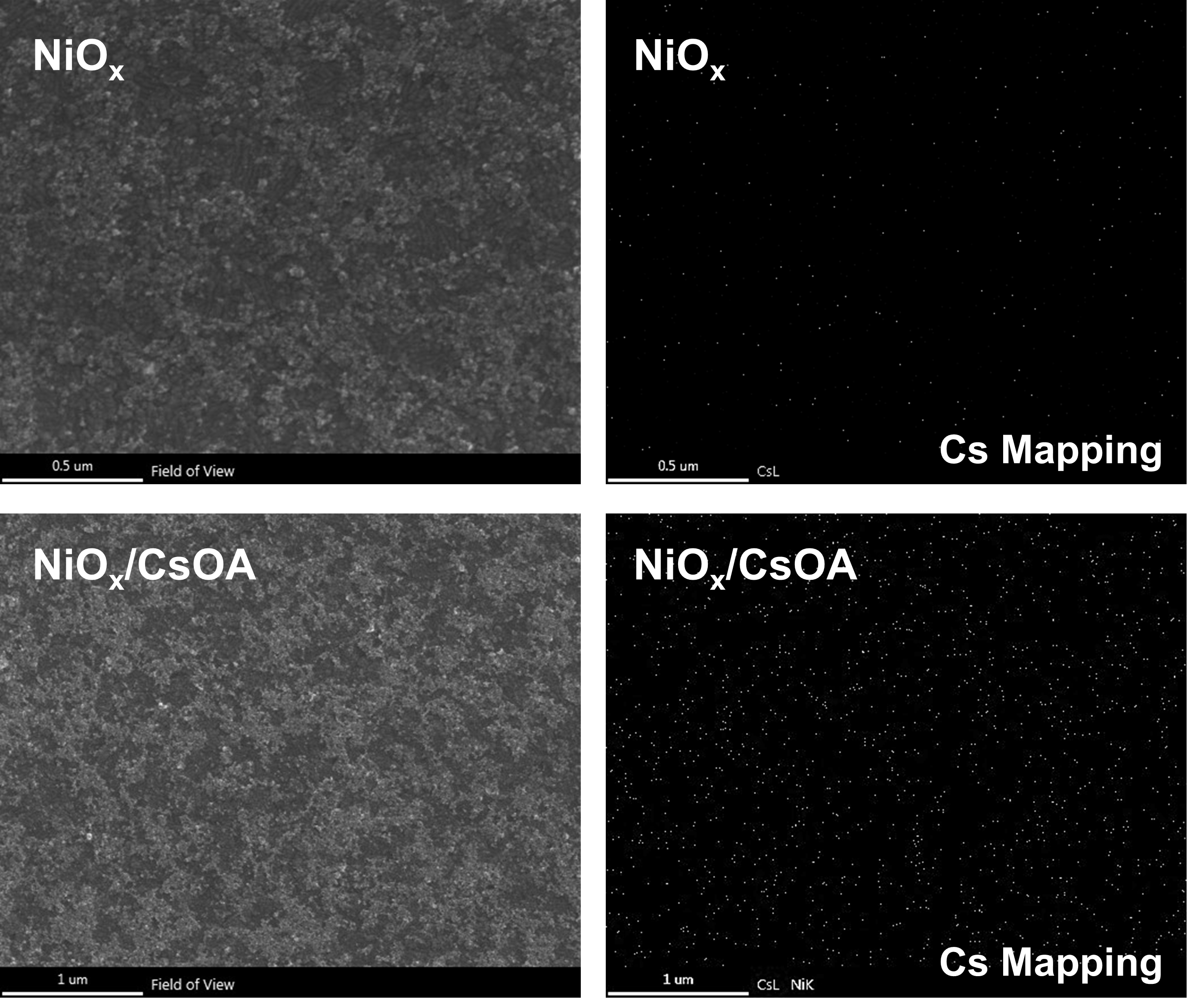
**

**Figure S1.** The SEM images of the NiO_x_ films and NiO_x_/CsOA and the corresponding energy dispersive spectroscopy (EDS) mapping.


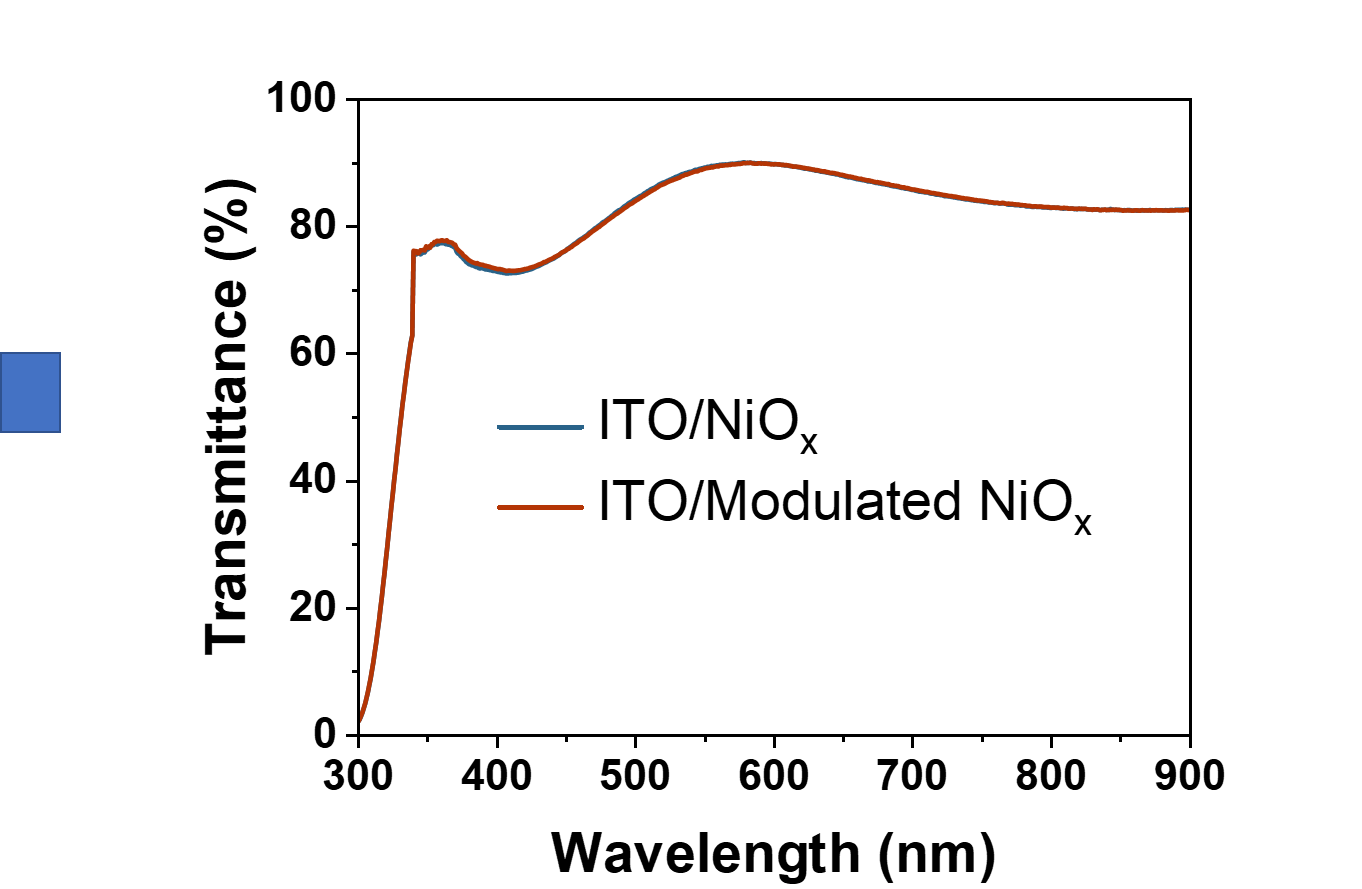


**Figure S2.** The transmittance of NiO_x_ and modulated NiO_x_.

*
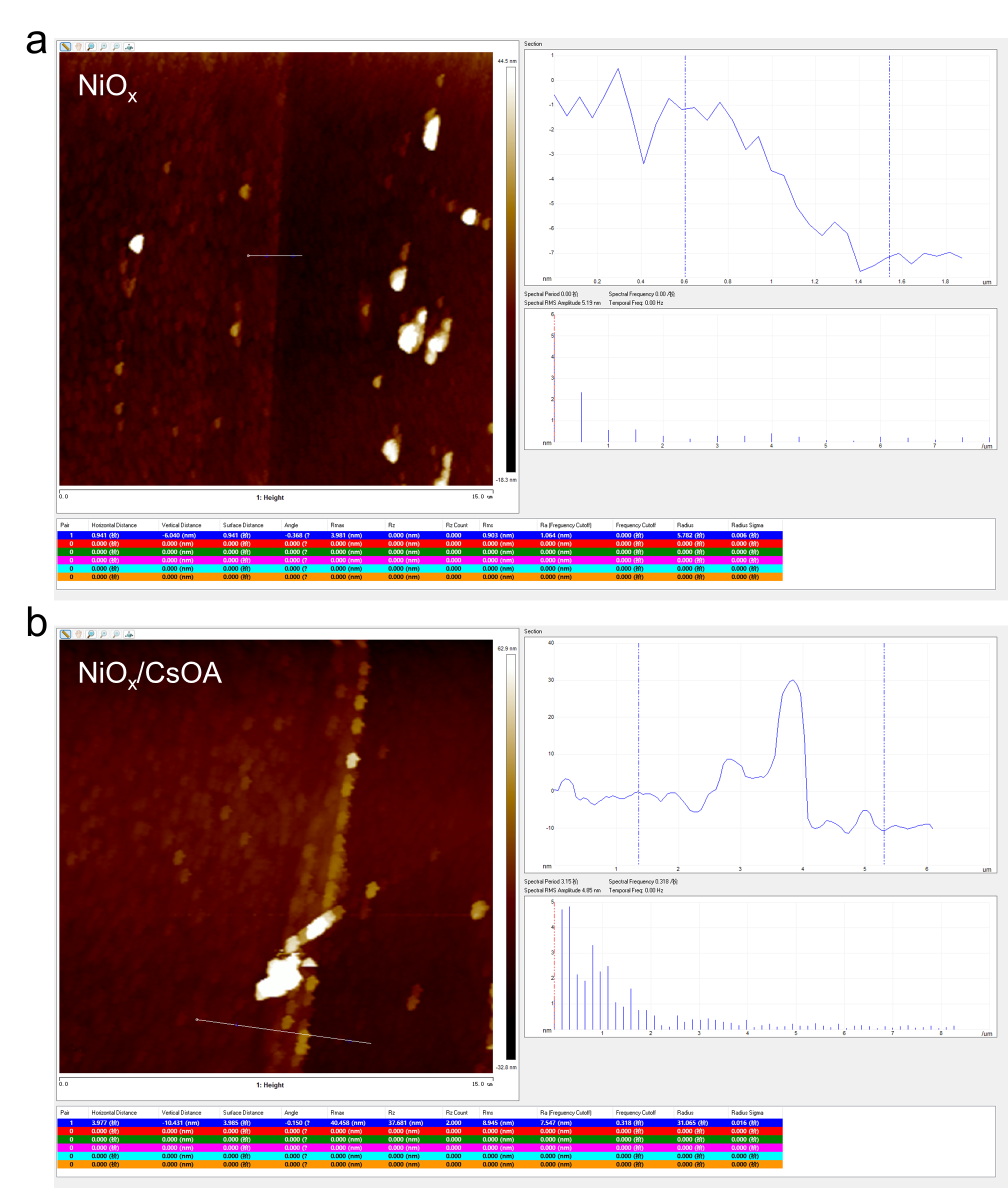
*

**Figure S3**. Tapping-mode AFM height images (15 × 15 μm) showing that the thickness of CsOA is about 4.4 nm (T_CsOA_=T_NiOx/CsOA_ - T_NiOx_=10.4 - 6.0=4.4 nm).


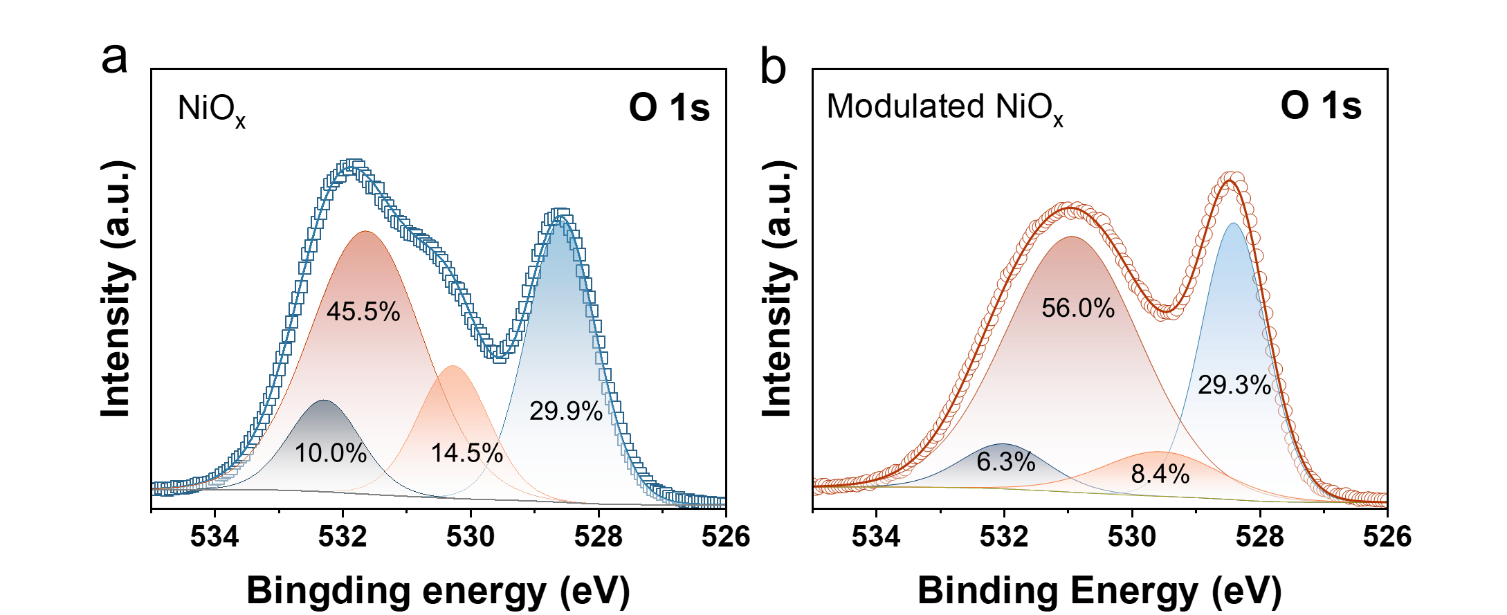


**Figure S4.** XPS spectra of O 1s for (a) NiO_x_ and (b) modulated NiO_x_.


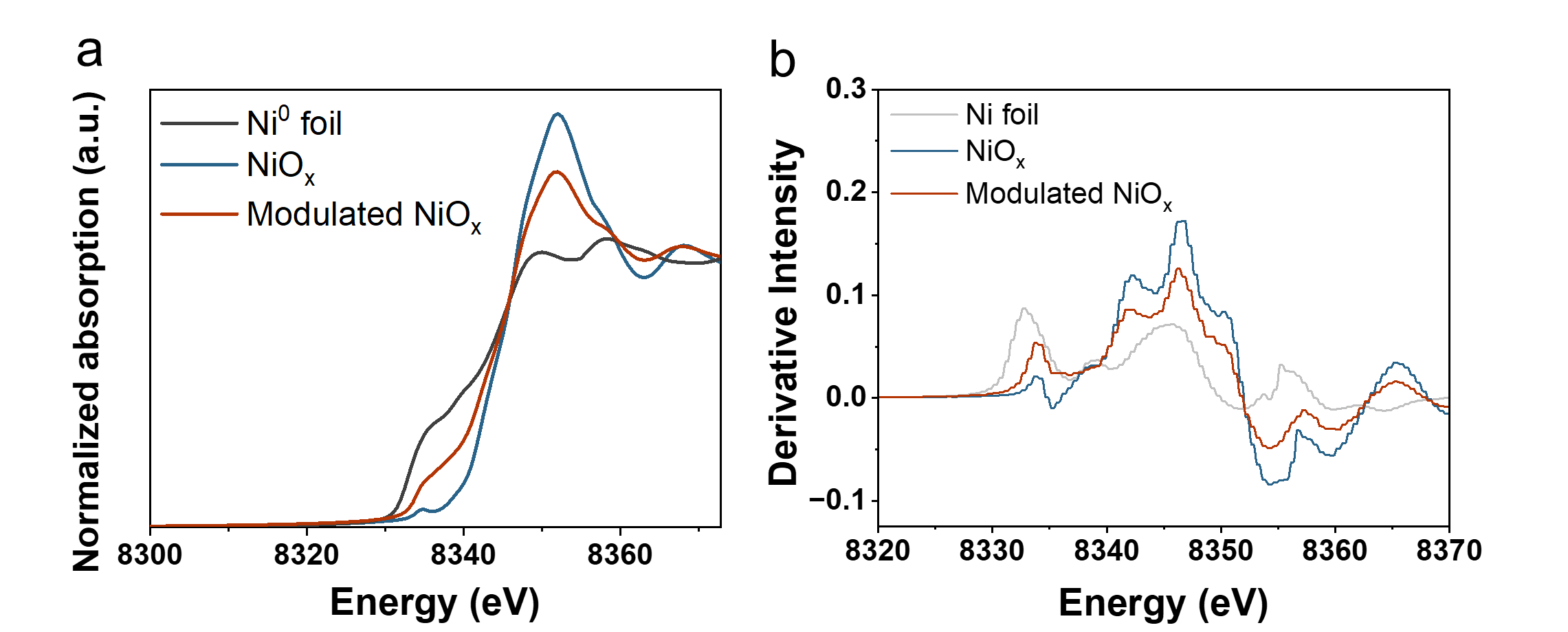


**Figure S5.** (a) Normalized XANES spectra and (b) magnitude of the Fourier-transformed EXAFS spectra of Ni_0_ foil, NiO_x_ and Modulated NiO_x_.

**Figure S6.** The conductivity of NiO_x_ (blue) and modulated NiO_x_ (red) films.


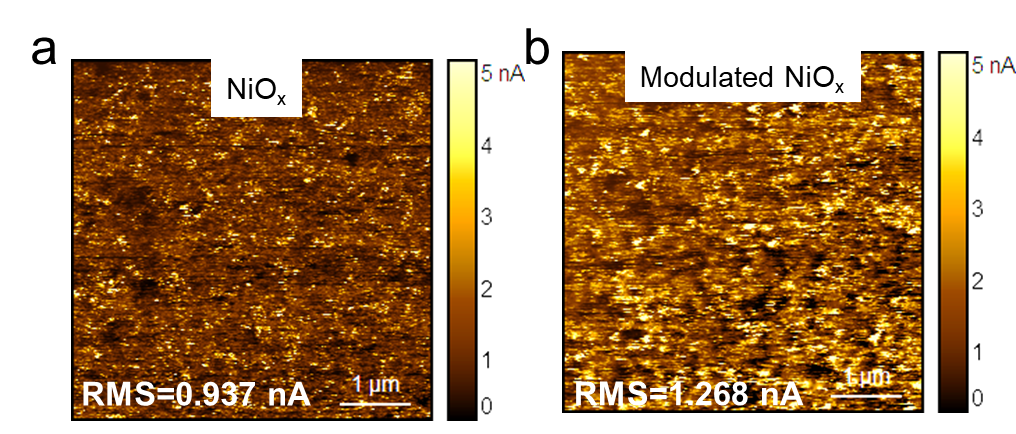


**Figure S7.** c**-**AFM images of (a) NiO_x_ and (b) modulated NiO_x_ films.

**
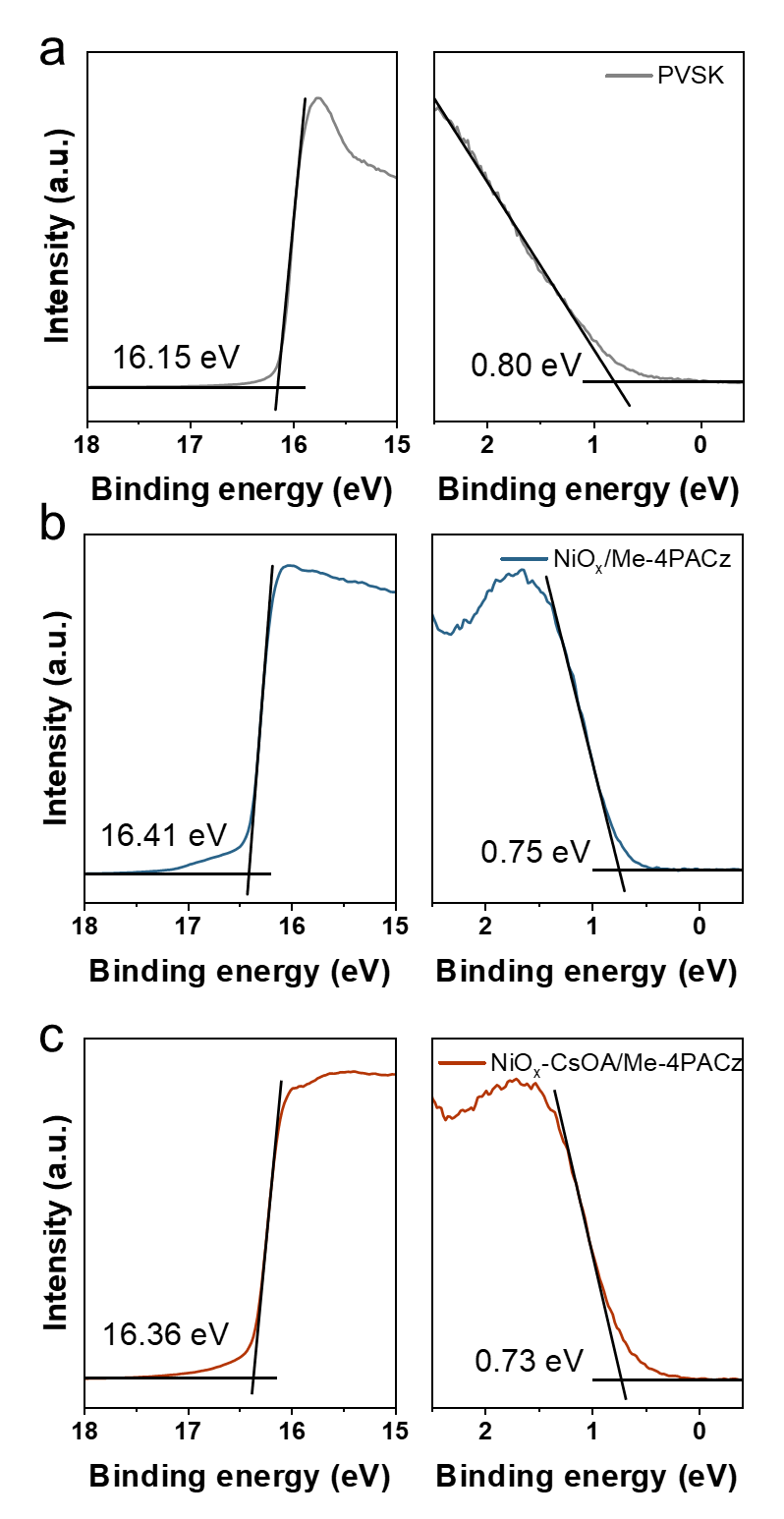
**

**Figure S8.** UPS spectra collected from (a) perovskite film, (b) ITO/NiO_x_/Me-4PACz (control), and (c) ITO/NiO_x_/CsOA–Me-4PACz (modified).


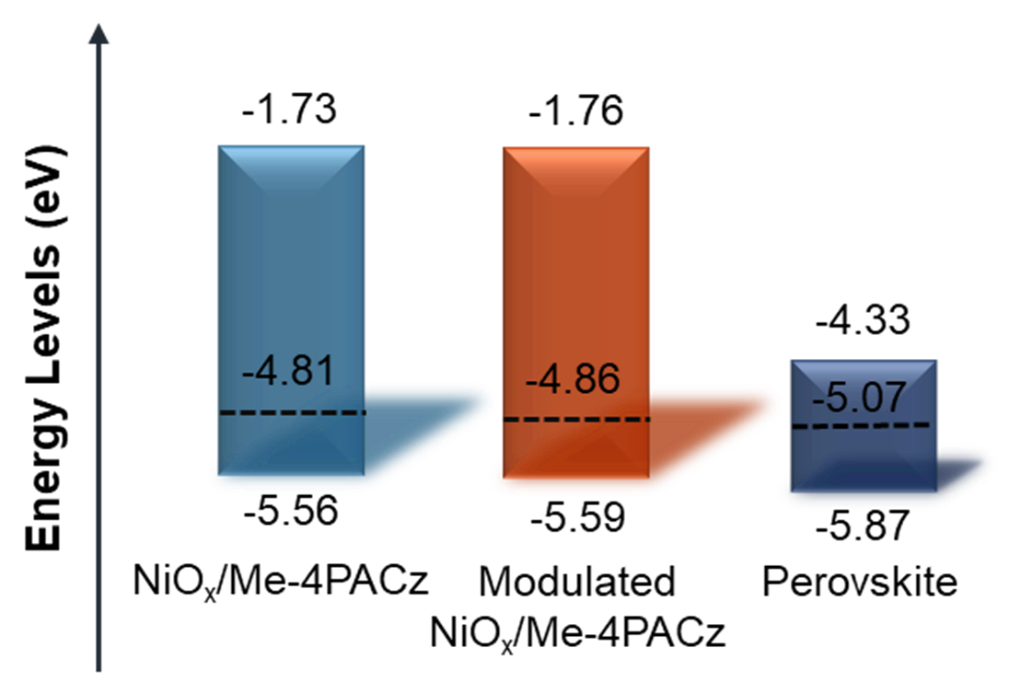


**Figure S9.** The schematic diagram of the energy level arrangement of ITO/NiO_x_/Me-4PACz, ITO/modulated NiO_x_/Me-4PACz, and Perovskite film.


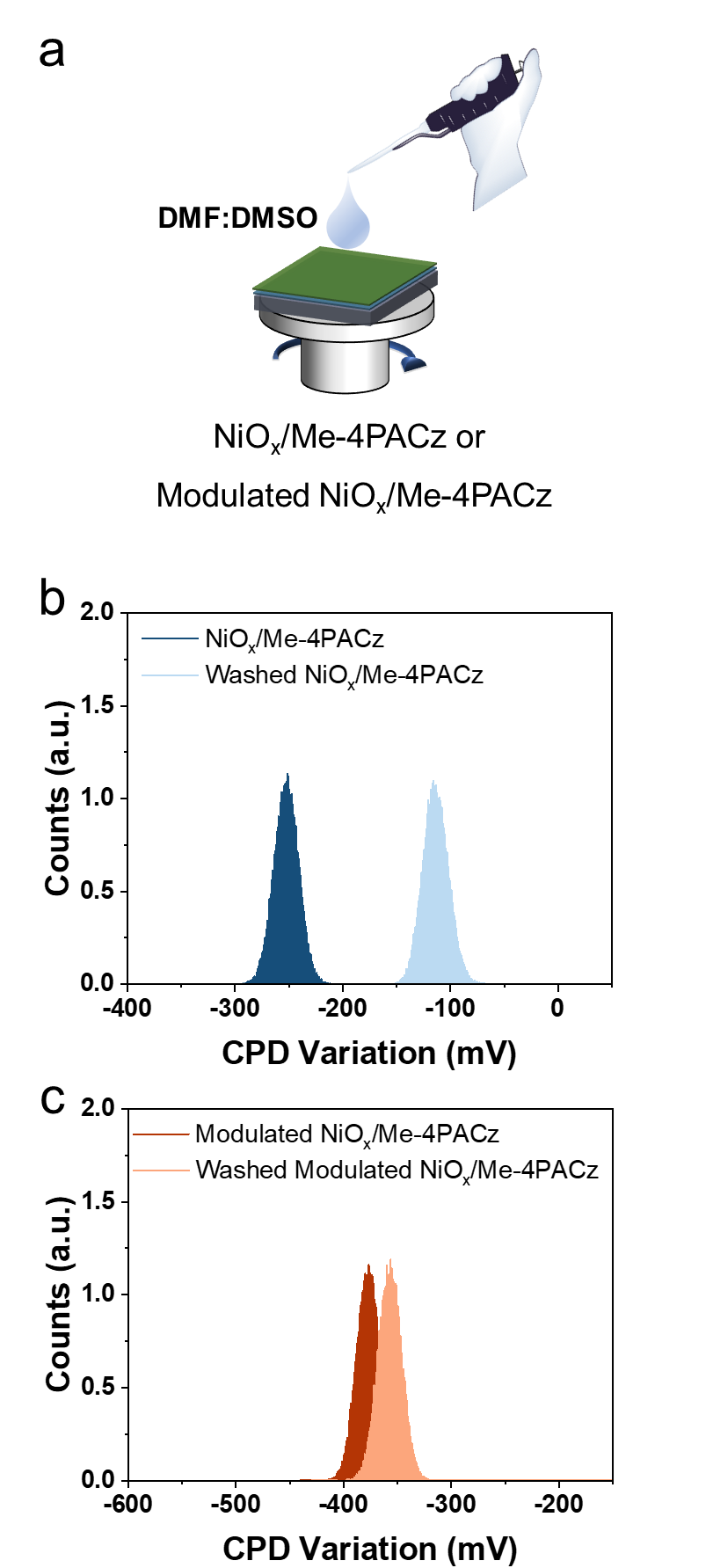


**Figure S10.** (a) Schematic diagram of substrate surface cleaning by DMF: DMSO=4:1. Contact potential difference (CPD) distributions of (b) NiO_x_/Me-4PACz and (c) modulated NiO_x_/Me-4PACz before and after washing.


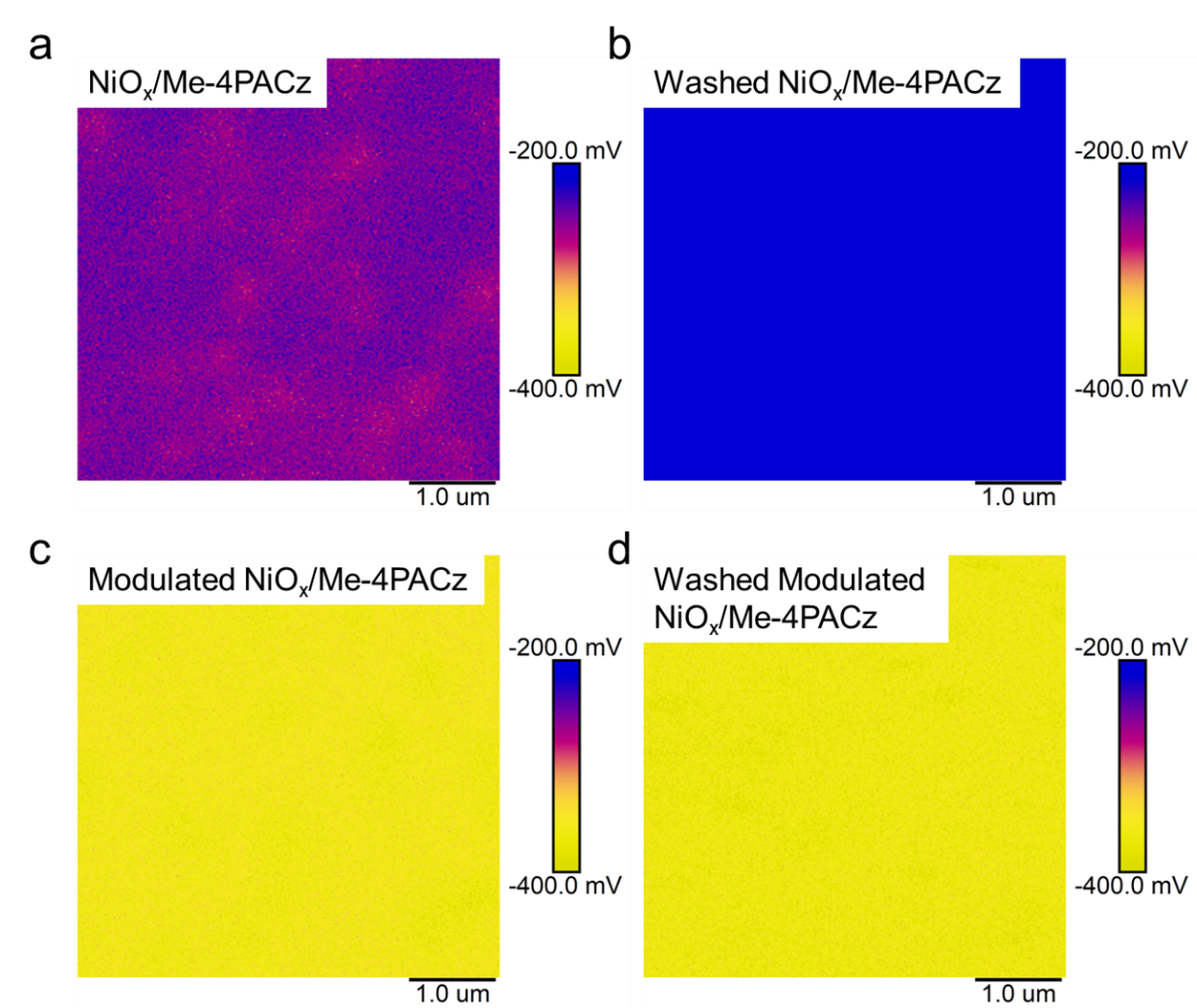


**Figure S11.** The KPFM images of (a) NiO_x_/Me-4PACz, (b) solvent-washed NiO_x_/Me-4PACz, (c) modulated NiOₓ/Me-4PACz and (d) solvent-washed NiOₓ/Me-4PACz.


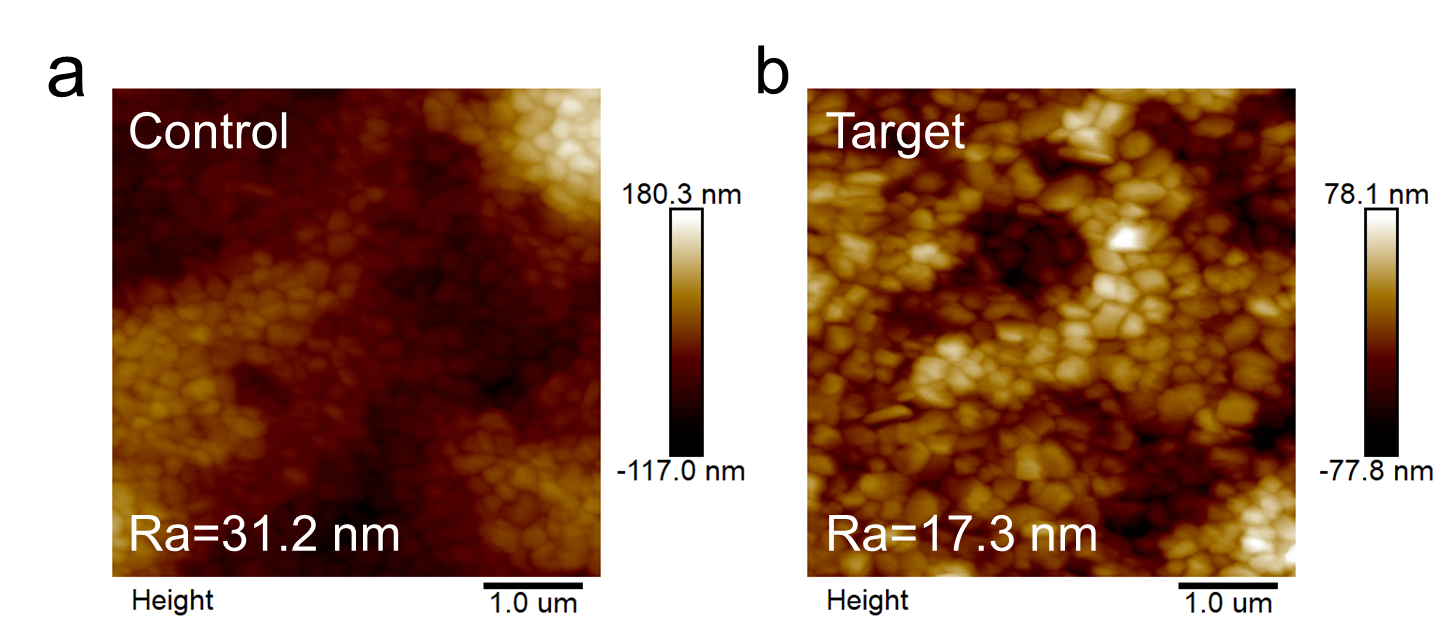


**Figure S12.** AFM images of (a) control and (b) target perovskite films.

**Figure S13.** The XRD spectra of control and target perovskite films.


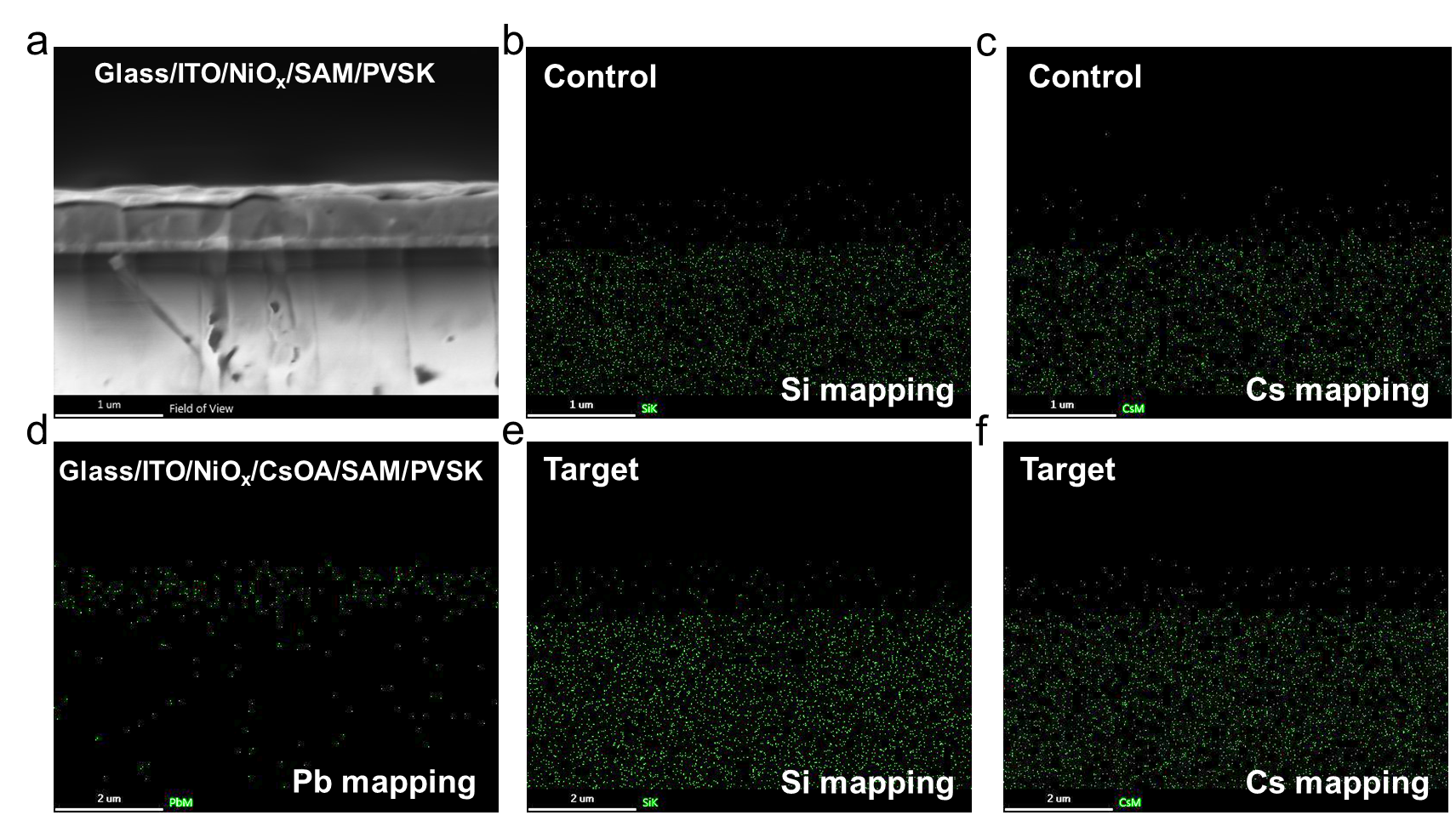


**Figure S14.** The cross-section SEM images of the perovskite (FAPbI_3_) films on (a-c) ITO/NiO_x_/SAM (control) and (d-f) ITO/NiO_x_/CsOA–SAM (target) and the corresponding energy dispersive spectroscopy (EDS) mapping.

**Figure S15.** The *in-situ* PL max intensity of control and target perovskite films.


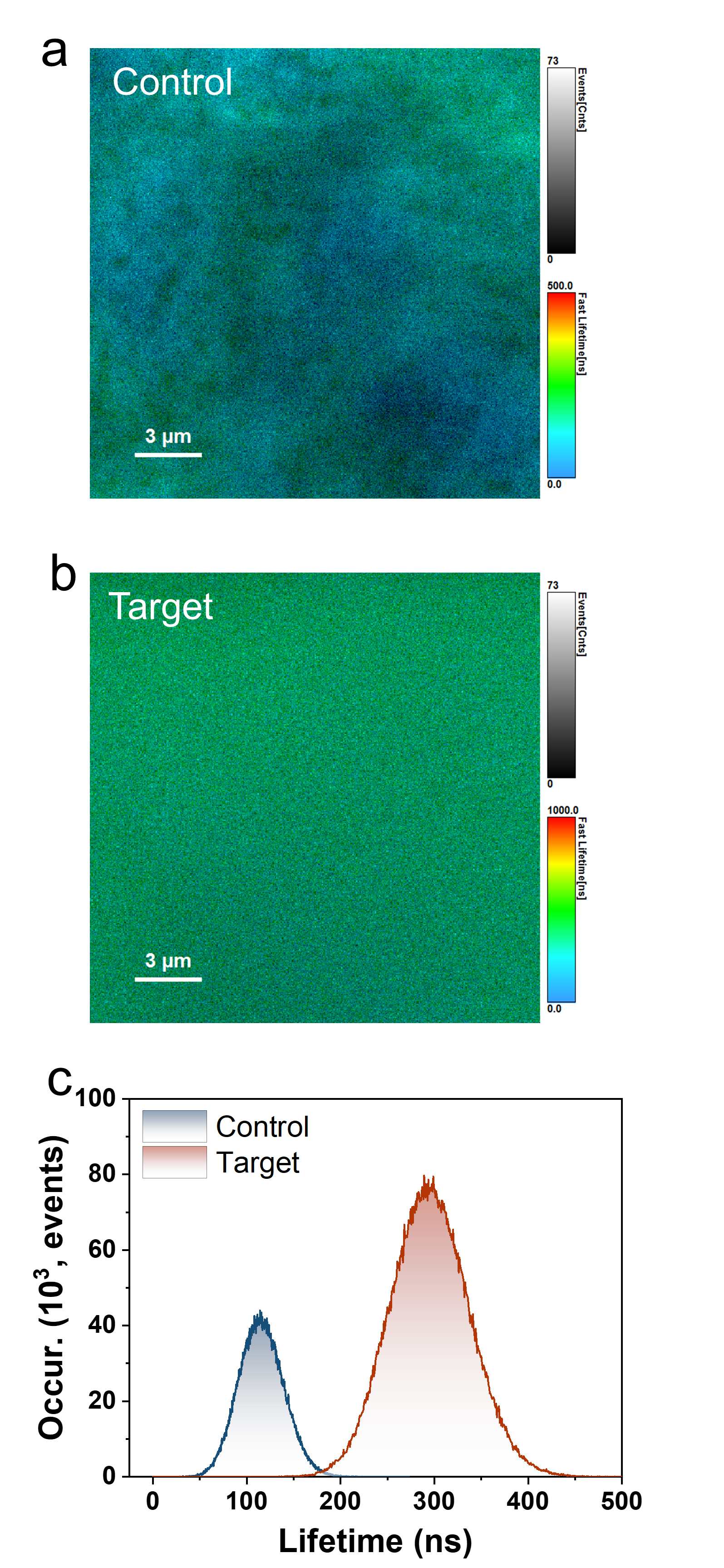


**Figure S16.** The TCFM image of the (a) control film and (b) target film. (c) The PL distribution of different carrier lifetimes for the corresponding films

**Figure S17.** XPS spectra of I 3d for control and target perovskite films.


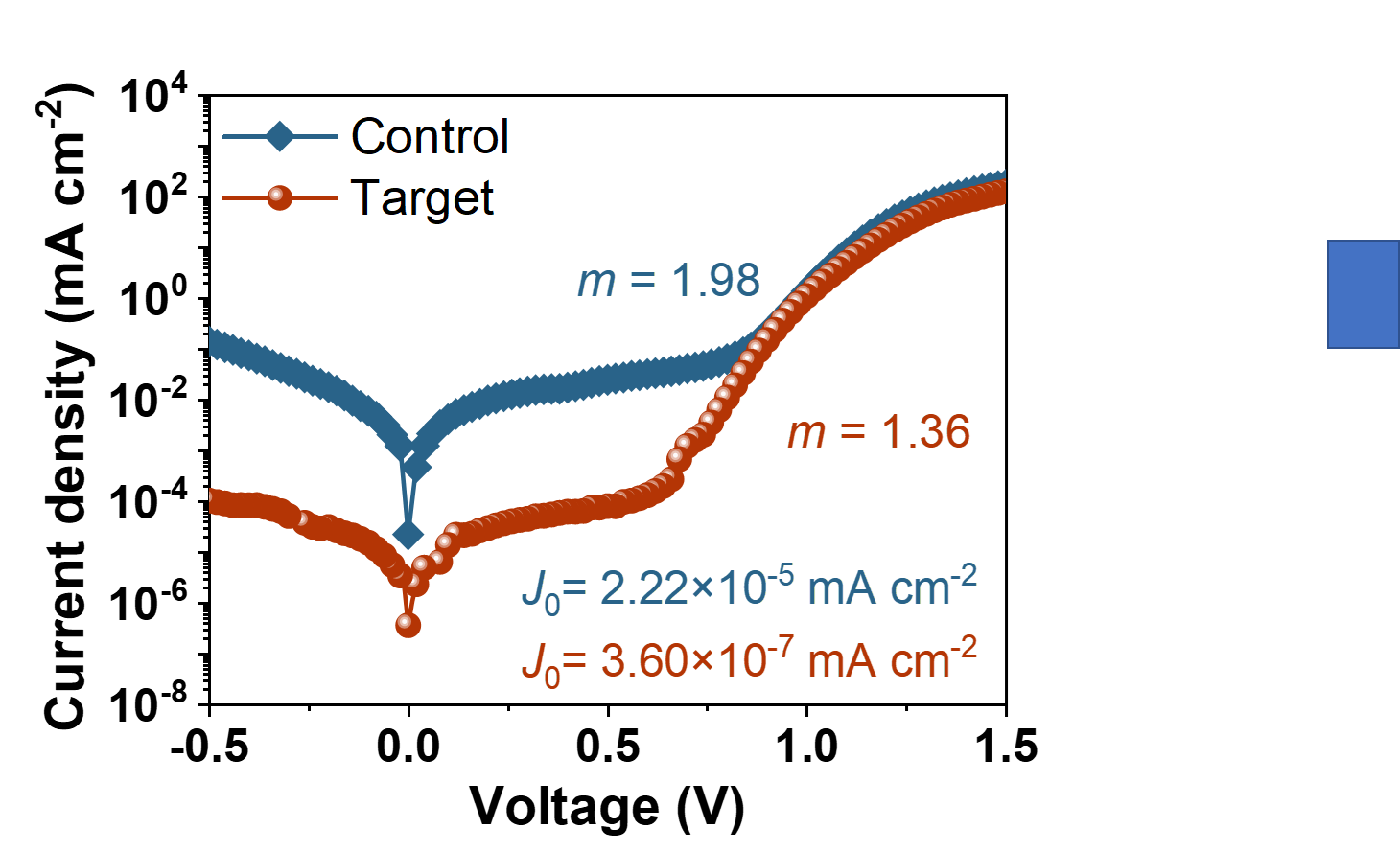


**Figure S18.** Dark *J−V* curves of the control and target devices.


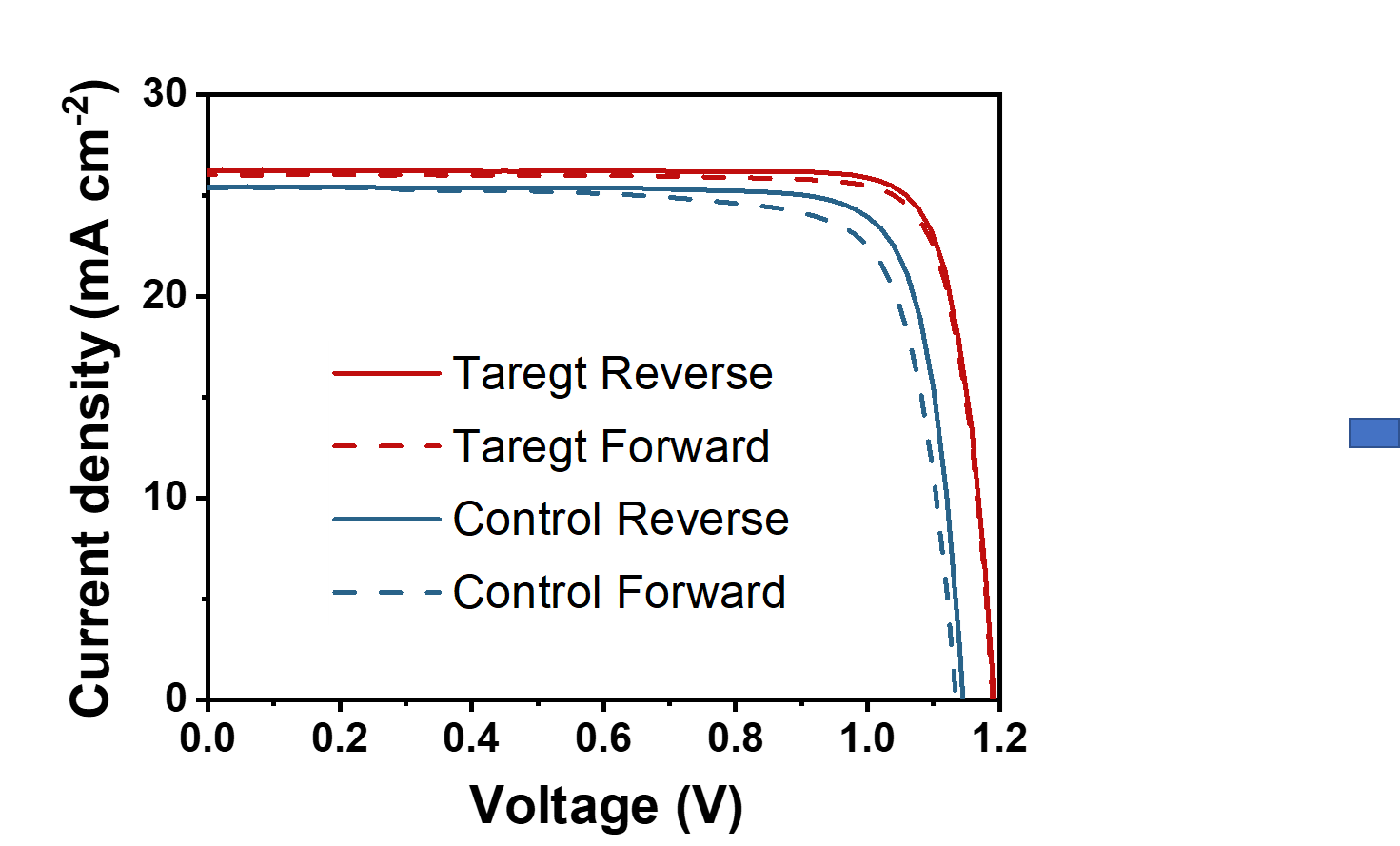


**Figure S19.** Reverse and forward scan *J-V* curves of control and target devices.

**Figure S20.** Stabilized PCE and current density of the champion control device determined by MPP for 600 s.


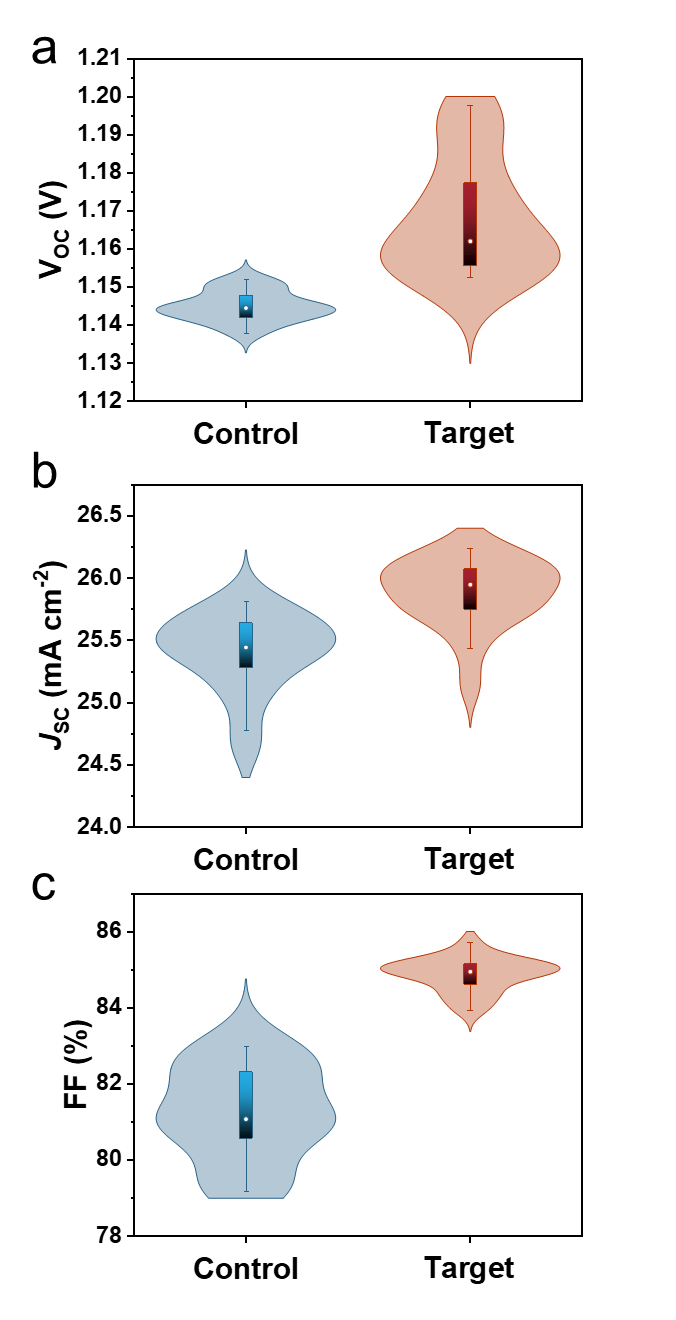


**Figure S21.** Statistical photovoltaic parameters of (a) *V_OC_*, (b) *J_SC_* and (c) *FF* of control and target PSCs.


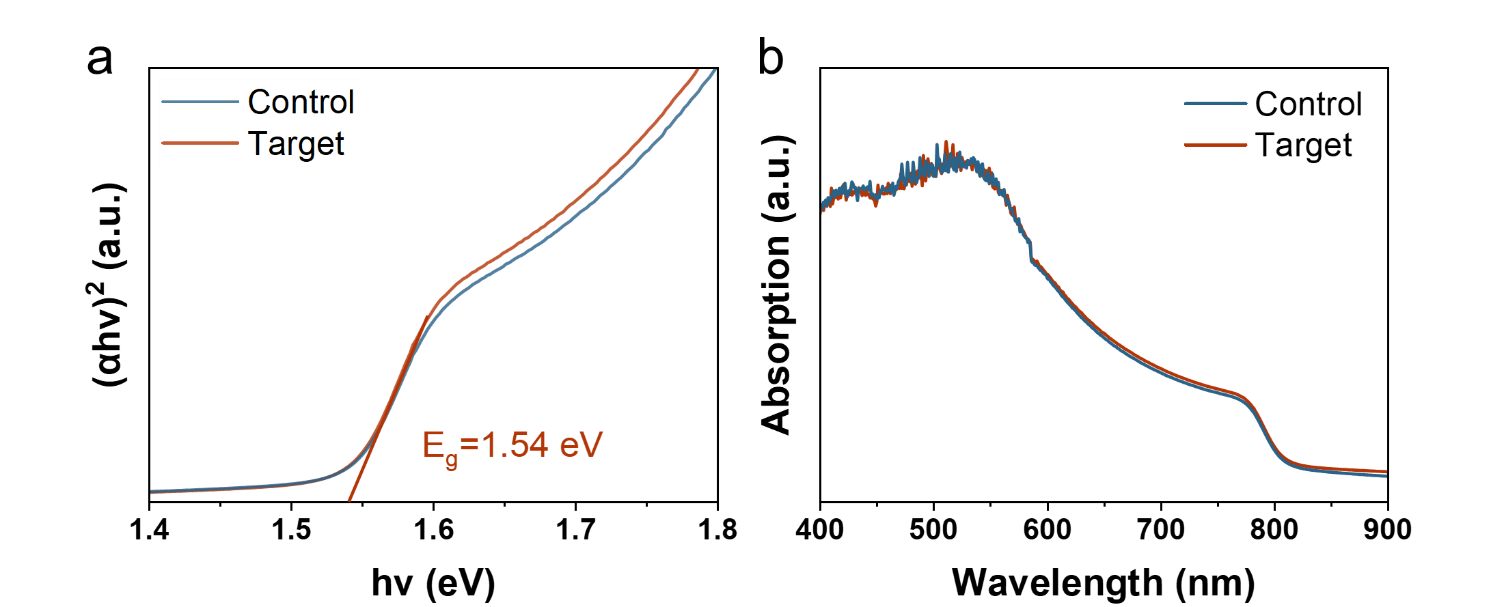


**Figure S22.** (a) Tauc plot and (b) UV-vis absorption spectrum for control and target perovskite films.


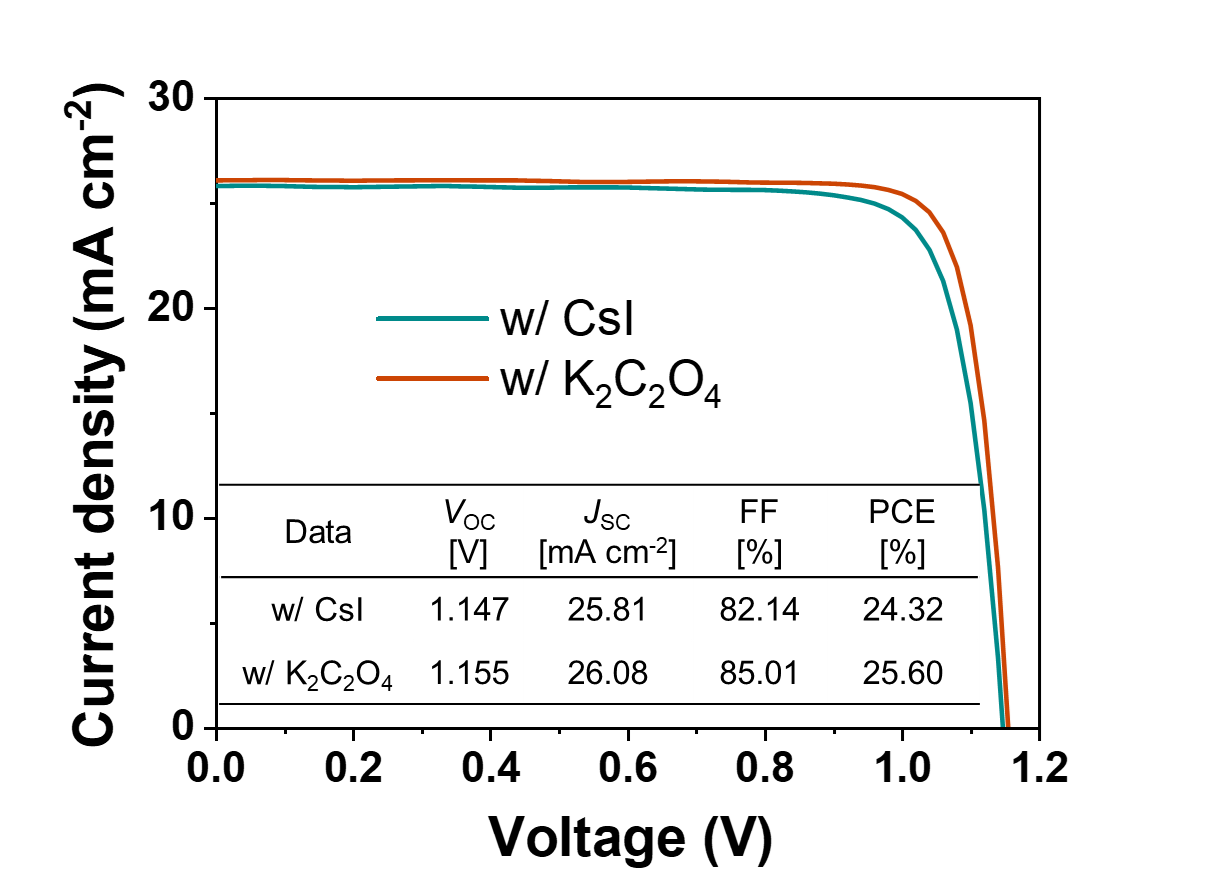


**Figure S23.** The *J-V* curves based on the modulated NiO_x_/Me-4PACz by CsI (w/CsI) or K_2_C_2_O_4_ (w/ K_2_C_2_O_4_).


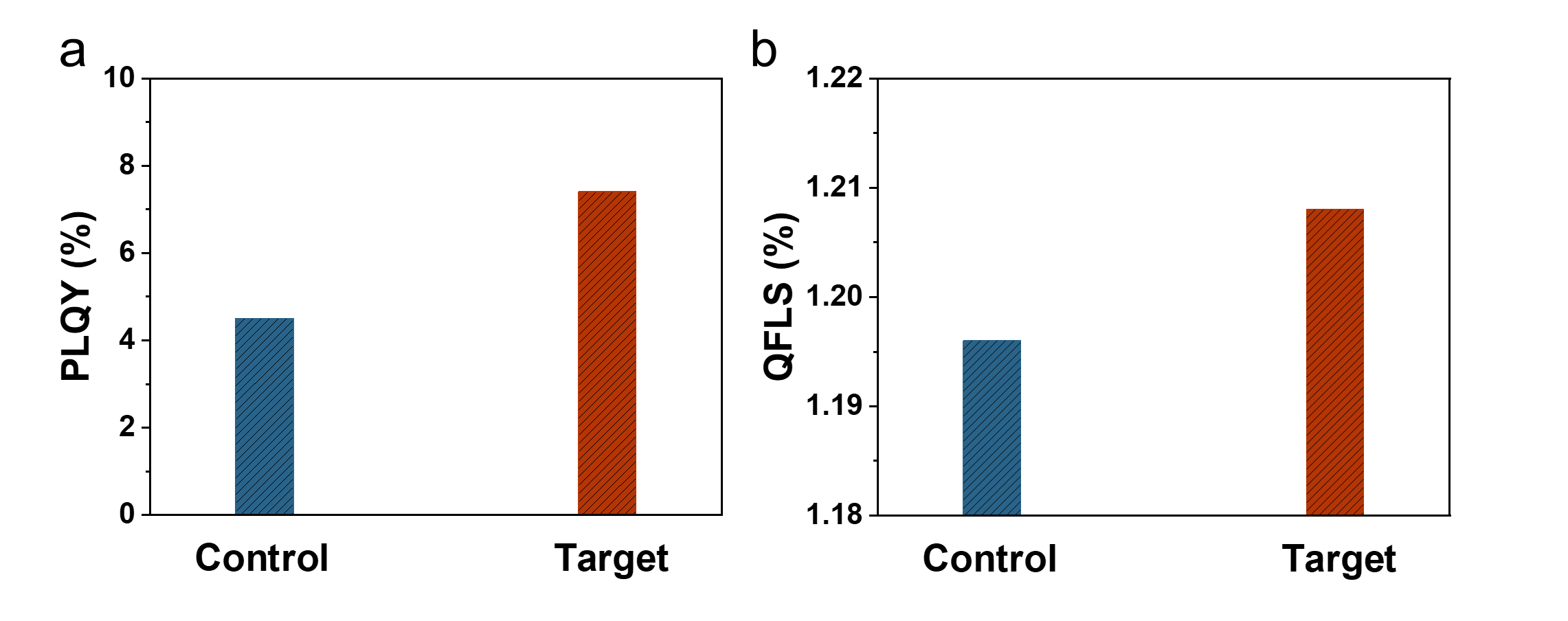


**Figure S24.** (a) Photoluminescence quantum yield (PLQY) for bare perovskite thin-films on ITO/NiO_x_/Me-4PACz (Control) and ITO/modulated NiO_x_/Me-4PACz (Target). (b) The quasi-Fermi level splitting (QFLS) of the same samples calculated from the PLQY presented in (a).

*
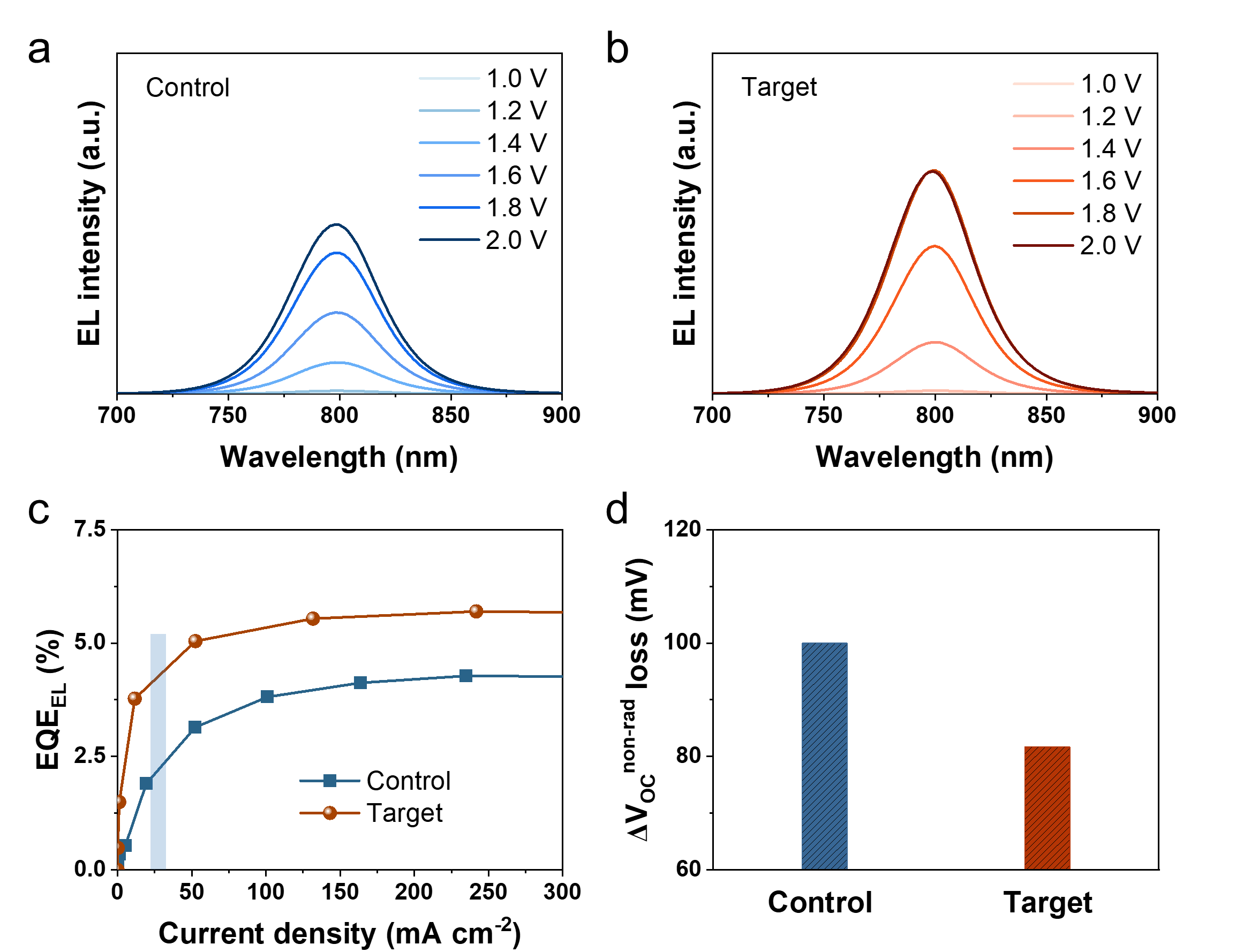
*

**Figure S25.** Electroluminescence spectra from (a) ITO/NiO_x_/Me-4PACz and (b) ITO/modulated NiO_x_/Me-4PACz-based PSCs operated as LEDs. EQE_EL_ as a function of the injection current density for PSCs based on (c) ITO/NiO_x_/Me-4PACz (Control) and ITO/modulated NiO_x_/Me-4PACz (Target) when operating as a light-emitting diode (LED). (d) The non-radiative recombination photovoltage loss (ΔV_OC_^non-rad^) of the same samples calculated from the EQE_EL_.


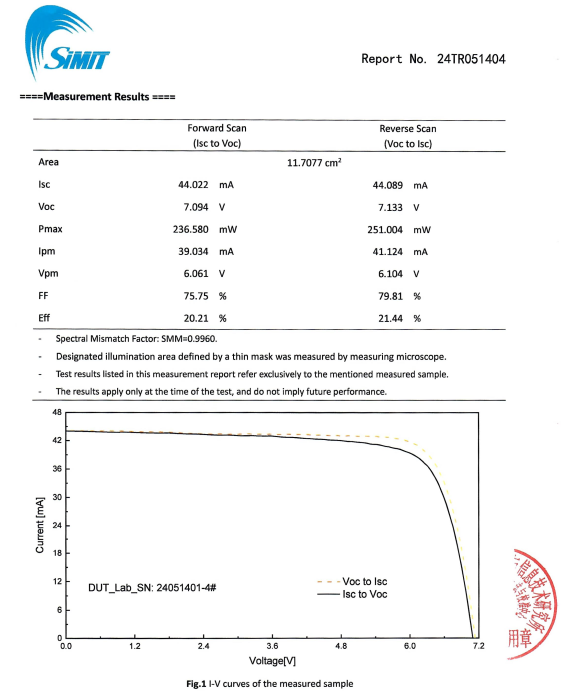


**Figure S26.** The certification report for inverted modules with an aperture area of 11.7077 cm^2^ from the Shanghai Institute of Microsystem and Information Technology, Chinese Academy of Sciences (SlMlT). The certified aperture-area PCE is 21.44% under reverse scan (short-circuit current (I_SC_) of 44.089 mA, *V*_OC_ of 7.133 V and FF of 79.81%), which is equal to an active-area PCE of 22.81% with an active area of 11.0 cm^2^ and a GFF of 94%.

**Table S1**. The Ni oxidation states were estimated from X-ray photoemission spectroscopy (XPS) spectra of NiO_x_ and Modulated NiO_x_.

|  | **NiO_x_** | **Modulated NiO_x_** |
| --- | --- | --- |
| NiO | 0.2395 | 0.245 |
| Ni(OH)_2_ | 0.172 | 0.1573 |
| Ni^3+^ | 0.4174 | 0.5467 |
| Ni^4+^ | 0.1712 | 0.0511 |
| Average valence | +2.76 | +2.65 |

**Table S2**. Photovoltaic performance parameters of the best control and target PSCs under different scan directions.

| **Samples** | **Scan**  **direction** | ***V_OC_***  **(V)** | ***J_SC_***  **(mA cm^-2^)** | ***FF***  **(%)** | **PCE**  **(%)** |
| --- | --- | --- | --- | --- | --- |
| Control | Reverse | 1.145 | 25.43 | 82.25 | 23.95 |
|  | Forward | 1.134 | 25.39 | 78.58 | 22.62 |
| Target | Reverse | 1.192 | 26.22 | 84.73 | 26.48 |
|  | Forward | 1.190 | 26.02 | 84.07 | 26.03 |

**Table S3**. Photovoltaic performance parameters of the PSCs based on the varying concentration of SAM on NiO_x_

| Concentration (mg mL^-1^) | V_OC_  (V) | *J*_SC_  (mA cm^-2^) | FF  (%) | PCE  (%) |
| --- | --- | --- | --- | --- |
| 0.5 | 1.151 | 25.43 | 81.39 | 23.82 |
| 1 | 1.148 | 25.69 | 80.69 | 23.80 |
| 2 | 1.144 | 25.78 | 80.93 | 23.87 |
| 3 | 1.137 | 25.24 | 81.21 | 23.30 |
| 5 | 1.135 | 24.98 | 80.13 | 22.71 |
